# Identification and functional studies of microbial volatile organic compounds produced by Arctic flower yeasts

**DOI:** 10.3389/fpls.2022.941929

**Published:** 2023-01-05

**Authors:** Jingjing Niu, Xuhuan Li, Siyu Zhang, Yifeng Yao, Yongping Zhang, Yixuan Liu, Xiaoya Peng, Jun Huang, Fang Peng

**Affiliations:** ^1^ China Center for Type Culture Collection (CCTCC), College of Life Sciences, Wuhan University, Wuhan, China; ^2^ State Key Laboratory of Systematic and Evolutionary Botany, Institute of Botany, Chinese Academy of Sciences, Beijing, China

**Keywords:** MVOC, flower, nectar, yeasts, Arctic

## Abstract

Microbial volatile organic compounds (mVOCs) can serve as a communication channel among microorganisms, insects and plants, making them important in ecosystem. In order to understand the possible role of mVOCs in Arctic ecology, the microbes in Arctic flowers and their mVOCs and effects on plants were investigated. This study aims to isolate different yeast species from the flowers of five Arctic plant species and further to explore the function of mVOCs emitted by these microbes to plant. It was found that the composition and amount of mVOCs produced by the isolated yeasts were considerably affected by changes in incubation temperature. When the incubation temperature rose, the species of alcohols, aldehydes, esters, organic acids, and ketones increased, but substances specific to low temperature decreased or disappeared. When yeasts were co-cultured with *Arabidopsis thaliana* without any direct contact, mVOCs produced by the isolated yeasts inhibited the seed germination of *A. thaliana* at low temperatures; however, the mVOCs promoted the chlorophyll content, fresh weight, root weight and flowering rate of *Arabidopsis* plants. Although the overall growth-promoting effect of yeast mVOCs was higher at 20°C than at 10°C, the growth-promoting effect on roots, flowers and chlorophyll was highest at 10°C. When cultured at 10°C, the mVOCs produced by *Cystofilobasidium capitatum* A37, *Cryptococcus* sp. D41, and *Sporidiobolus salmonicolor* D27 had the highest growth-promoting effects on the root, flowering rate and chlorophyll content of *Arabidopsis*, respectively. In the co-culture system, some new mVOCs were detected, such as hendecane, tetradecane, and 1-hexanol that have been proven to promote plant growth. In addition, mVOCs of the isolated Arctic yeasts could inhibit the growth of several microorganisms, especially filamentous fungi. It was the first time to prove that mVOCs produced by the isolated yeasts had varying effects on plant growth at different incubating temperatures, providing a reference for the interactions between microorganisms and plants and their possible responses to climate change in the Arctic area. Moreover, the characteristics of promoting plant growth and inhibiting microbial growth by mVOCs of Arctic yeasts would lay a foundation for potential applications in the future.

## 1 Introduction

Microbial volatile organic compounds (mVOC) are produced and released during microbial metabolism with the characteristics of low molecular weight metabolites (< 300Da), low polarity and high vapor pressure (Werner et al., 2016). Till 2018, over 2000 kinds of mVOCs were identified and reported (Lemfack et al., 2018). mVOCs can be produced by microorganisms from different ecological environments (Polizzi et al., 2012; Jishma et al., 2017; Sobhy et al., 2018; Calvo et al., 2020; Elmassry et al., 2020; Wang et al., 2021). In recent years, several researchers have explored the properties of mVOCs, making them a research hotspot. mVOCs are known to serve as a communication channel among microorganisms, insects, and plants. For example, it was found that honey bees preferentially foraged on *Asaia astilbes*-inoculated nectars, which means the mVOCs emitted by *A. astilbes* could attract pollinators, likely leading to a higher visiting rate and consequently a higher seed set of plants (Rering et al., 2020). However, mVOCs act as repellent signals for nutrition thieves (Sobhy et al., 2018). Furthermore, the mVOCs of different microbes played varied parts in ecosystem. For example, the mVOCs of *Saccharomyces cerevisiae* represented critical signal in attracting fruit fly *Drosophila melanogaster*, while the common bacterium *Gluconobacter* sp. reduced pollination success (Becher et al., 2012; Vannette et al., 2013). This may be because of the difference in the composition of mVOCs between bacteria and yeast (Rering et al., 2018).

Former studies on the effects of mVOCs on plants focused on nectar habitats. The nectarines inside the flower have the ability to secrete nutrient-rich liquid, known as nectar. Nectar is mainly composed of sugar, amino acids, and trace substances (Nicolson and Thornburg, 2007). Therefore, it serves as a good source of nutrients for the growth of microorganisms, especially in oligotrophic Arctic regions. Owing to the movement of wind and the visiting of insects, a variety of microbes, including bacteria, yeast, and filamentous fungi, may exist on the surfaces of and inside the flowers. However, the high concentration and osmotic pressure in the nectar niche limit the colonization of different kinds of microorganisms; therefore, only some special microorganisms grow and reproduce in the flowers. In recent years, studies have demonstrated that the VOCs emitted by nectaric yeasts change nectar properties (such as nectar temperature, pH, sugar concentration) and exert influences on plants, such as thermal effect, pollination effect, and reproductive effect (Herrera and Pozo, 2010; Álvarez-Pérez et al., 2012; Canto and Herrera, 2012; Herrera et al., 2013; Rering et al., 2018; Sobhy et al., 2018; Pozo et al., 2019; Poveda, 2021).

Previous studies have focused on mVOCs emitted by flower yeasts in the temperate zone or subtropical zone, little was studied of that in extremely cold habitats, such as the Arctic region. The temperature of the Arctic region has increased at the rate of approximately 1°C per decade (IPCC, 2013), and this continuously rising temperature may influence the metabolism of flower microbes, the growth of plants, and the behavior of plant visitors. Therefore, a changed microbial metabolism would change the ecosystem of polar area, consequently. In order to explore the possible roles of mVOCs in Arctic ecosystems, the objectives of this study were: first to isolate and identify yeast species from flowers of five Artic species, second to analyze the effect of VOCs emitted by those microbes on plant germination and growth at different temperatures, and finally to identify the volatile organic compounds produced by the microbe and in the plant-microbe system.

## 2 Materials and methods

### 2.1 Isolation and identification of yeast strains from Arctic flowers

Ny-Ålesund is the northmost area in Svalbard Archipelago, Arctic. The atmosphere, ocean, glacier and sea level in this region are the sensitive indicators and recorders of climate change. Despite the environmental conditions in Ny-Ålesund were harsh (strong ultraviolet radiation, extremely low temperature and drought), a variety of plants, animals and microorganisms still exist. The native plants, such as *Silene acaulis* and *Cerasticum arcticum*, are capable of flowering (Yao et al., 2014). In order to adapt to the rugged environment, the plants are usually small-sized and grow closer to the surface of naked ground or rock. The summer in Ny-Ålesund is short and cool, and the temperature ranges from -8°C to 20°C, with the average temperature being 8.5°C. Therefore, plants should perceive the temperature changes sensitively and act quickly to complete the reproduction stage—just as Mondoni et al. (2015) stated, ‘extremely rapid growth is one of the characteristics of Arctic vegetation. 

**Figure 1:**
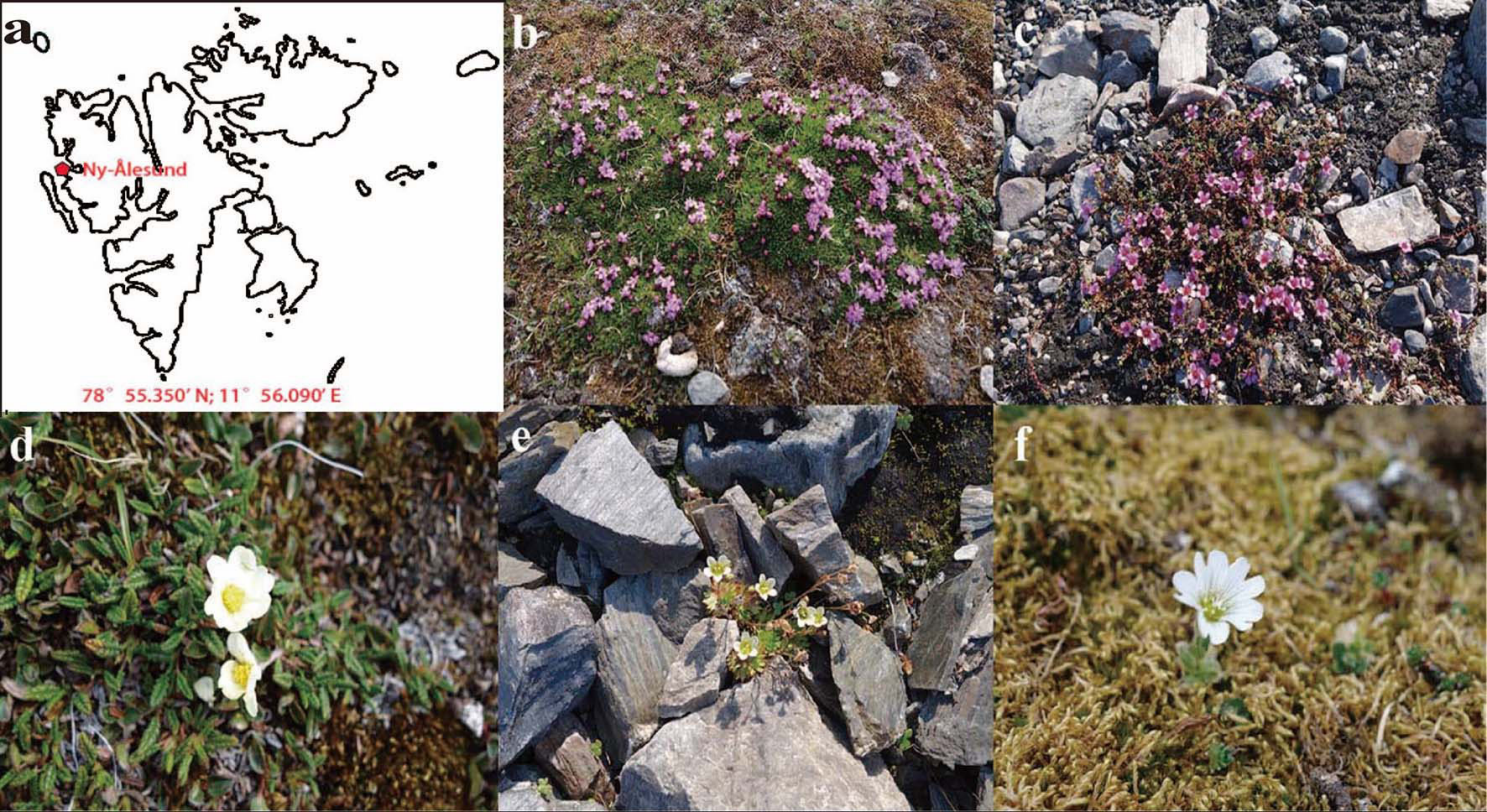
Location of Ny-Ålesund and flower samples. **(A)** Location of Ny-Ålesund, Svalbard Archipelago **(B–F)** Flower samples; **(B)** *Silene acaulis*; **(C)** *Saxifraga oppositifolia*; **(D)** *Dryas octopetala*; **(E)** *Saxifraga cespitosa*; **(F)** *Cerasticum arcticum*.

In this study, five flower samples were collected into Eppendorf tubes through a scissor that had been pre-sterilized by 75% ethanol from five blooming plants in Ny-Ålesund, Svalbard Archipelago (78°55.350’ N; 11°56.090’ E), the Arctic in July 2019. The plants were *Silene acaulis*, *Saxifraga oppositifolia*, *Dryas octopetala*, *Saxifraga cespitosa*, and *Cerasticum arcticum*. The sampling site and flowers were provided in **Figure 1**. After sampling, the flowers were transferred as soon as possible to the Chinese laboratory at low temperature. Under laboratory conditions, samples were soaked in sterile 0.85% (w/v) NaCl, mixed fully by using a vortex mixer, and diluted serially to varying concentrations. Then, 100 µL of these dilutions were added to per Petri plate (diameter: 9 cm) that filled with potato dextrose agar, soybean sprout extract glucose medium, YMA (Yeast-Malt extract Agar) containing 10% fructose, YMA containing 10% glucose and YMA containing 20% glucose. The soybean was purchased from a nearby supermarket, and all the other reagents were purchased from Sigma. All treatments were performed with three biological replicates (n=3). After spreading evenly, the Petri dishes were incubated at 10°C for about one month until single colonies were formed. Yeast identification was performed by using conventional morphological procedures and molecular evolutionary studies. Total genomic DNA was extracted by thermal cracking. Subsequently the 26S rDNA, ITS rDNA and 18S rDNA fragments were amplified using NL1/NL4, ITS1/ITS4 and NS1/NS8 primers, respectively (Baturo-Ciesniewska et al., 2017; Jiang et al., 2021). After purification on 1% agarose gel, the PCR products were sequenced by Tianyi Huiyuan Biotech Co., Ltd. (Wuhan, Hubei, China). Then the sequence would be aligned on the NCBI GenBank database to confirm the taxonomy.

### 2.2 mVOCs collection, identification and analysis

The difference in mVOCs production after the yeasts were incubated at varying temperatures was evaluated. Because of the lack of data on the nectar composition of plants in Ny-Ålesund, the nectar survey results of previous studies (Kessler and Baldwin, 2007; Lievens et al., 2015; Rering et al., 2018; Pamminger et al., 2019) were referred. As a result, 6% sucrose, 12% fructose, 12% glucose, 0.5% peptone, 0.3% yeast extract and 0.3% malt extract were used to mimic nectar and this culture medium was called ‘artificial nectar medium’. Fourteen isolated yeasts (**Table 1**, except for strain *Metschnikowia reukaufii*) were cultured in artificial nectar medium at 4°C, 10°C and 20°C until they reached the late exponential stage. Although high temperature can elevate the efficacy of mVOCs adsorption, the cell of yeast would rupture under high temperature, and some VOCs that not produced during metabolizing would release, affecting the results. Hence, the supernatant of yeast culture was used in mVOCs absorption stage to make results more rigor. After centrifuged, and the supernatant (about 5 ml) was transferred to a 20 mL light-proof mason jar for adsorption.

**Table 1:** Detailed information about selected yeasts.

Strains	Closest relative species(highest % identity)	Coverage of ITS/26S(%)	Identity of ITS/26S(%)	Flower origin	Preservation number
A29	*Tausonia pullulans*	100	100.00	*Silene acaulis*	CCTCC SY2019479
A211	*Cystofilobasidium* sp.	99	100.00	*Silene acaulis*	CCTCC SY2019480
A37	*Cystofilobasidium capitatum*	99	100.00	*Silene acaulis*	CCTCC SY2019400
A38	*Debaryomyces hansenii*	100	100.00	*Silene acaulis*	CCTCC SY2019401
C21	*Vishniacozyma victoriae*	99	100.00	*Dryas octopetala*	CCTCC SY2019414
C23	*Rhodotorula mucilaginosa*	100	100.00	*Dryas octopetala*	CCTCC SY2019415
C31	*Microbotryum silenes-acaulis*	98	99.85	*Dryas octopetala*	CCTCC SY2019416
D13	*Goffeauzyma gilvescens*	99	100.00	*Saxifraga cespitosa*	CCTCC SY2019417
D24	*Mrakia gelida*	100	100.00	*Saxifraga cespitosa*	CCTCC SY2019482
D27	*Sporidiobolus salmonicolor*	99	99.83	*Saxifraga cespitosa*	CCTCC SY2020010
D41	*Cryptococcus* sp.	99	98.77	*Saxifraga cespitosa*	CCTCC SY2019420
E24	*Cystobasidium laryngis*	100	99.84	*Cerasticum arcticum*	CCTCC SY2020012
731	*Mrakia blollopis*	100	99.68	*Saxifraga cespitosa*	CCTCC SY2015731
MR	*Metschnikowia reukaufii*	99	99.81	*Aconitum piepunense*	CCTCC SY2014008

CCTCC (China Center for Type Culture Collection, Wuhan, Hubei, China).

Before the adsorption stage, 200 ppm dimethyl formamide (internal reference) was added to every aquiform sample. Then the solid-phase microextraction fiber (75 µm, 1 cm, carboxen/polydimethylsiloxane; Supelco, Bellafonte, PA, USA) was inserted and exposed to the headspace of the hermetical mason jar through the silicone septa. Subsequently, the mVOCs evaporated into the headspace would be adsorbed onto the fiber. This reaction was accomplished in a water bath at a temperature of 60°C for 6 hours. Finally, the mVOCs were thermally desorbed in the injection port at 250°C in splitless mode into a 450GC-320MS (Varian; USA) outfitted with an Agilent J&W HP-5 column (30 m × 0.25 mm × 0.25 µm). Helium was used as a carrier gas with a permanent flow rate of 1 mL/min. And the oven temperature ramp settings were as follows: the initial GC oven temperature was 45°C for 5 min, which was raised from 45°C to 180°C at a rate of 10°C/min, then to 230°C at a rate of 20°C/min and kept at 230°C for 5 min, finally to 250°C at a rate of 20°C/min and kept at 250°C for 5 min. The transfer line temperature and ion source temperature were 260°C and 200°C, respectively. The energy was 70 eV in electron impact mode and the MS data were obtained in full-scan mode with a m/z range of 30–800. To identify metabolites, we used the NIST database by matching the mass spectrum and retention index. Because the GC-MS instrument used in this study cannot calculate the peak area automatically, the peak area can only be calculated through its inherent function by man. Subsequently, the peak area of single substance was calculated and the relative amount was defined using the following formula:


$$\text{Relative amount}=\frac{{\text{S}}_{\left(\text{single substance}\right)}}{{\text{S}}_{\left(\text{internal reference}\right)}}$$

Where S means peak area (unit).

Every treatment was performed with three biological replicates (n=3). The data were shown in a heatmap by using the GraphPad Prism software. And data analysis was accomplished *via* Veen diagram in the website http://bioinformatics.psb.ugent.be/webtools/Venn/.

### 2.3 Antimicrobial effect of mVOCs *in vitro*

The effect of mVOCs produced by 14 isolated yeast strains (**Table 1**) were tested on several microbes to test whether these mVOCs affect the growth of other microbes. The effects of mVOCs produced by 14 yeast strains were tested *in vitro* on several microbes, including bacteria (*Escherichia coli*, *Pseudomonas putida*, *Pseudomonas aeruginosa*, *Erwinia oleae* and *Erwinia tasmaniensis*), yeast (*Candida albicans*), and filamentous fungi (*Penicillium expansum*, *Penicillium polonicum*, *Penicillium commune*, *Mucor racemosus*, *Aspegillus fumigatus*, *Aspergillus* sp., *Fusarium* sp. and *Trichoderma* sp.). All tested microorganisms were acquired from China Center for Type Culture Collection (CCTCC), Wuhan, Hubei, China. The fungi were identified as described in 3.1, while bacteria were identified as described earlier (Zhang et al., 2021). The culture mediums of the tested microorganisms were listed in **Table S1**. Methods used in previous studies was referred (Rouissi et al., 2013; Calvo et al., 2020), and all 14 yeasts (**Table 1**) were involved. Specifically, two petri plates with same size were used, however, these two plates contained different culture mediums and consequently for different microorganisms incubation. For the inoculation of tested microbes, an agar plug (diameter: 0.5 cm) from actively-growing of fungal culture plate (3 days-old culture on PDA at 20°C) was placed at the center of PDA plate. And if the tested microbe was bacteria, a piece of sterile filter paper (diameter: 0.5 cm) filled with 100 µL fresh culture (2 days-old culture in corresponding medium at 20 °C) was added at the center of corresponding culture medium, instead. For yeast inoculation, the culture suspension (10^8^ cell/mL) was spread evenly in artificial nectar plates. Then, these two different plates were sealed with double parafilm in a mouth-to-mouth way (the upper plate was the plate inoculated with tested microbe, the nether plate was the plate inoculated with yeast) and incubated at 20°C for about 5 d. Water was used as control and all treatment were performed with three biological replicates (n=3). Afterwards, the fungal diameter was measured and compared with control plates (without yeast inoculation). The results were expressed as the percentage of inhibitory rate (%) in comparison with the control using the following formula, which means the higher the inhibition rate value was, the stronger inhibitory effect was.


$$\text{Inhibition rate}=\frac{\left({\text{D}}_{\text{control}}-0.5)-({\text{D}}_{\text{treatment}}-0.5\right)}{{\text{D}}_{\text{treatment}}-0.5}\times 100\%$$

Where D means colony diameter

Data were subjected to one-way analysis of variance (ANOVA) followed by comparison of multiple treatment (mVOCs of different yeast strain) with control (non-mVOCs), using *post hoc* Fisher’s Least Significant Difference (LSD) test to determine the significant difference at 5% level of significance.

### 2.4 Effect of mVOCs onseed germination

In order to explore how the mVOCs emitted by different yeasts influence the seed germination at different incubation temperature, the experiment was conducted. In this study, seed germination rates at varying incubation temperatures of 4°C, 10°C and 20°C were tested to explore the effects of mVOCs of 14 yeast strains (**Table 1**) on seed germination. *Arabidopsis thaliana* is the model plant in plant-VOC interaction research (Hung et al., 2013), therefore, *A. thaliana* Colombia-0 (eco-type) seeds were used in this study and bought from the website https://arabidopsis.info/CollectionInfo?id=134.

Two different sizes of Petri plates were used in this experiment (**Figure S1**), specifically, the smaller Petri plate (diameter: 9 cm) was placed in the center of the larger Petri plate (diameter: 14 cm). The smaller plate filled with artificial nectar medium (about 20 ml per plate) was used for yeast growth, while the larger one filled with 1/2 MS medium supplemented with 1% surcose (pH=5.8, about 60 ml per plate) was used for seed cultivation. The seeds were surface-sterilized in a bleach solution (20% sodium hypochlorite solution with 0.1% Triton X-100) for 6 min. After washed with sterile water for 3 times, 30 seeds were inoculated evenly on the surface of the plant culture medium, which was filled with the space between smaller plate and the larger plate. After the yeast and seeds were inoculated, the plates were sealed with double parafilm to prevent mVOCs leakage. Sterile water inoculated in the smaller plate was used as blank control, and all treatments as well as blank control were performed with three biological replicates (n=3). The plates were separately incubated at 4°C, 10°C and 20°C in dark until the seeds of blank control almost germinated. The germination rate was calculated by dividing the number of germinated seeds by the total number of seeds in the same plate. And the data were analyzed using the software SPSS Statistics version 25 to test the influence of different kinds of mVOCs mixture of yeasts to *A. thaliana* gemination, using Dunnett’s test to determine the significant difference at 5% level of significance.

### 2.5 Effect of mVOCs on *A. thaliana* growth

The influence of mVOCs emitted by 14 yeast strains to the growth of *A. thaliana* were tested. *A. thaliana* Colombia-0 (eco-type) seeds and 14 yeast strains (**Table 1**) were used in this experiment. 1/2 MS medium (Coolaber, Lot: PM1060) supplemented with 1% sucrose (pH=5.8) and artificial nectar medium were used for plant cultivation and yeast incubation, respectively. The methods were modified as per a study by Hung et al. (2013). Specifically, 10 µl fresh yeast culture was inoculated on the surface of artificial nectar (0.8 ml) in a single suspended Eppendorf tube (1.5 ml), and one surface-sterilized *A. thaliana* seed was inoculated on the surface of plant culture medium (about 20 ml per tube) at the bottom of one glass tube (height: 18 cm; diameter: 3 cm). After sealed with tube stopper and double parafilm, the mVOCs emitted by yeasts would release into the inside space of the closed glass tube and would exert influence to the growth of *A. thaliana* inside the same tube (see **Figure S2**). Sterile water inoculated on the surface of artificial nectar was used as a control. All treatments were performed with 10 biological replicates (n=10) and all tubes (yeast-plant co-culture tubes) were incubated at 20°C or 10°C with 16 h light and 8 h dark conditions.

After about 6-weeks (at 20°C) or 15-weeks (at 10°C) incubation, relevant parameters were measured. Fresh weight (including total fresh weight, shoot weight and root weight), shoot height and longest root length (hereinafter, root length) of *A. thaliana* were measured by using electronic weighing scale and vernier caliper, respectively. The determination and calculation of chlorophyll content (including total chlorophyll, chlorophyll a and chlorophyll b) were performed as described in previous studies (Hung et al., 2013; Zhou et al., 2018). Firstly, appropriate amount of plant leaves was collected and cut into pieces with scissors, then all pieces were immersed in 80% acetone solution and put still at 4°C overnight or longer until the leaves turn white. A_645_ and A_663_ were then measured using a microplate reader (Powerwave XS, BioTek, USA), and the chlorophyll content was calculated using the following formulas:


$$\text{Chlorophyll a content}=(12.72{\text{A}}_{663}-2.59{\text{A}}_{645})\times \text{v}/\text{w}\times 1,000$$

$$\text{Chlorophyll b content}=(22.88{\text{A}}_{645}-4.67{\text{A}}_{663})\times \text{v}/\text{w}\times 1,000$$

$$\text{Total chlorophyll content}=(20.29{\text{A}}_{645}+8.05{\text{A}}_{663})\times \text{v}/\text{w}\times 1,000$$

Where A means absorption; v means the volume of 80% acetone solution used; w means the weight of *Arabidopsis* leaves used.

The folds compared with control (for each parameter, the fold was calculated by dividing the mean of treatment by the mean of control) was processed *via* Excel (Microsoft, Redmond, WA) and shown in a heatmap by using the GraphPad Prism software.

### 2.6 Interaction between mVOCs and plant

To detect whether there was an interaction between plant and mVOCs while co-culturing, part of the co-culture systems (yeast-plant co-culture tubes) that incubated at 20 °C were involved in this experiment. The VOCs present in the co-culture system of plant and yeasts were collected and tested by microextraction and GC-MS, respectively. First, a small hole was drilled in the lid of the co-culture tube (without touch of the suspended Eppendorf tube and *A. thaliana*) and a pre-cleaned solid-phase microextraction fiber (75 µm, 1 cm, carboxen/polydimethylsiloxane; Supelco, Bellafonte, PA, USA) was inserted into it. The tube was placed vertically at room temperature (about 22°C) for 1.5 days, and then the fibers were taken to the GC-MS for analyte desorption, separation and detection, immediately. The adsorption conditions and operation program of GC-MS were performed the same as described in 2.2. All treatments were performed with three biological replicates and blank control (without yeast inoculation) were also involved in this experiment (n=3). Then, the information of detected VOCs (including the kind, retention time, peak area) would be acquired. Excluding the VOCs detected in blank control or yeast culture, the finial VOCs would be considered as new VOCs for they only be produced while and yeast interacting.

### 2.7 Effect of selected VOCs on *A. thaliana* growth

There might be some kinds of VOCs in the co-culture system of *A. thaliana* and yeasts, and these substances may play important role in affecting the growth of *A. thaliana*, hence, their effect were detected. After detection of the VOCs inside the yeast-plant co-culture system, three kinds of new VOCs were selected to test their effects on the growth of *A. thaliana* as described in a previous study (Wu et al., 2019). These selected VOCs were 1-hexanol (CAS: 111-27-3), hendecane (CAS: 1120-21-4) and tetradecane (CAS: 629-59-4). In this study, 10 µg, 100 µg, 500 µg, 1000 µg and 2000 µg doses per Petri plate were tested. First, all tested standards were serially diluted with ethanol. Next, three surface-sterilized *Arabidopsis* seeds were placed on one side of the divided Petri plate containing 1/2 MS medium supplemented with 1% sucrose, and a piece of sterile filter paper (1 cm×1 cm) filled with 100 µl different doses of pre-diluted solutions were placed on the opposite side of the divided plate. Then, the plates were sealed with double parafilm and put still at 20°C with the condition of 16 h light and 8 h dark for 3 weeks. Water was used as control and every treatment was performed with seven plates (n=7). Finally, all growth parameters of *A. thaliana* were used same as the methods described in 2.5. Statistical analysis was done by the software SPSS Statistics version 25, using Dunnett’s test with the significant difference at 5% level of significance to test the influence of different kinds of mVOCs mixture of yeasts to the growth of *A. thaliana*.

## 3 Results

### 3.1 Isolated yeasts from Arctic flowers

A total of 51 yeast strains belonging to 4 classes, 7 orders, 9 families, 11 genera and 12 species were obtained from five types of flowers. Among these strains, *Mrakia* was the most abundant genus, while the *Tausonia* was the least abundant genus (**Figure S3**) and *Tausonia* sp. only existed in *Silene acaulis* flowers. Among the five flower samples, the highest yeast diversity was observed in *Saxifraga cespitosa* flowers (7 genera). Thirteen isolated yeast strains belonging to different species were selected for further studies **Table 1** and it was found that these yeasts grew well at 4°C–20°C and a pH of 4.0–8.0. Moreover, they tolerated 5% (w/v) NaCl and grew well in high sugar medium—all yeasts grew well in 10%–40% sucrose, 10%–30% fructose, and 1%–40% glucose.

And all these 13 yeast strains would be used as experimental materials, furthermore, one classic nectaric yeast *Metschnikowia reukaufii* (MR), acquired from China Center for Type Culture Collection (Wuhan, Hubei, China), was used as a control in these experiments (**Table 1**). And all isolates were stored at China Center for Type Culture Collection.

### 3.2 mVOCs identification and analysis

**Figure 2:**
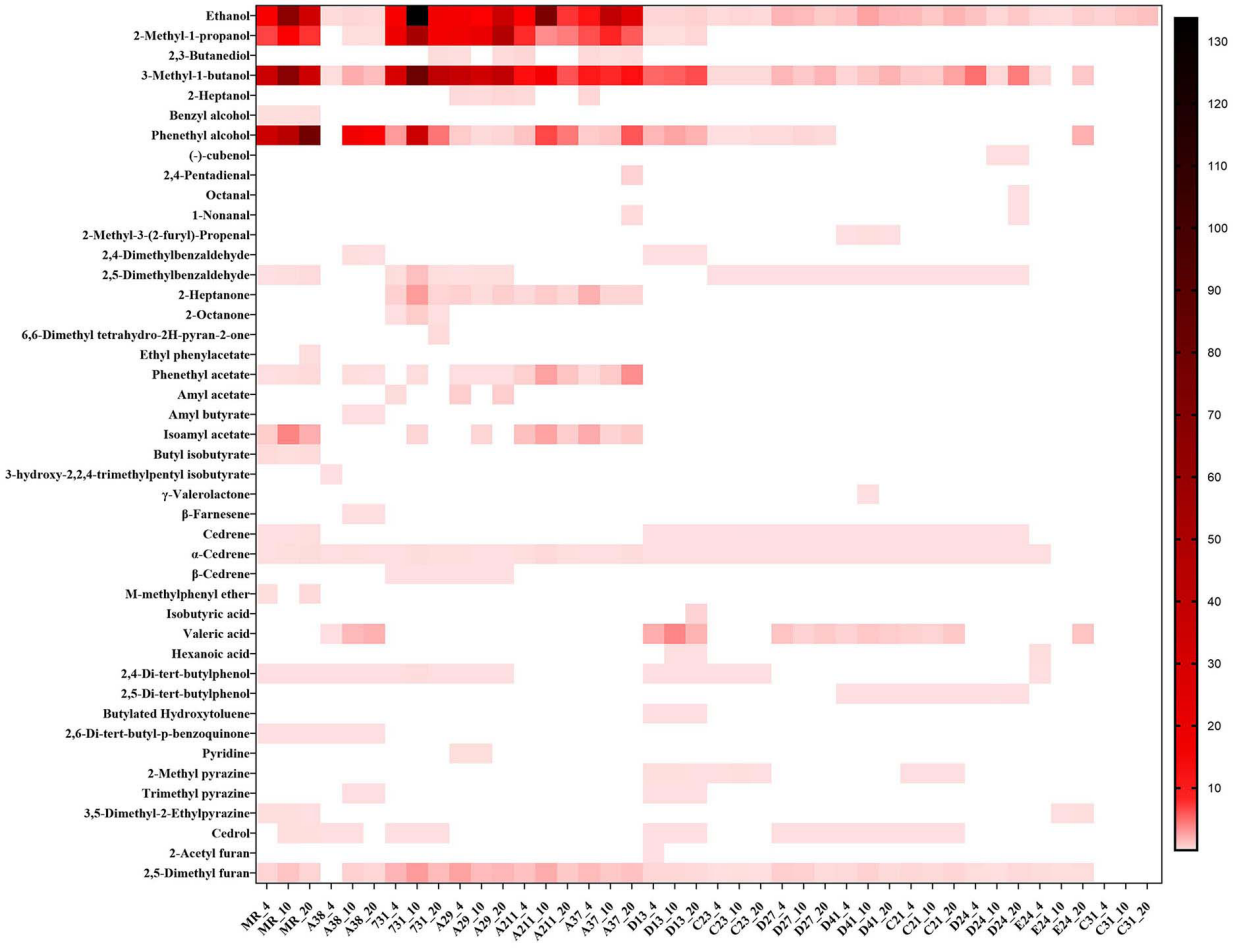
Composition of yeast mVOCs at varying temperatures. X-axis indicates yeast strains and incubation temperature; Y-axis indicates detected mVOCs; ‘_4’ means incubate under 4°C; ‘_10’ means incubate under 10°C; ‘_20’ means incubate under 20°C; n=3; different color means different relative abundance: the darker the color block was, the more relative abundance the material had.

By using microextraction and GC-MS analysis, 44 kinds of volatile substances were detected from 14 yeast strains grown at different temperatures. Detailed information, including the substance name and amount, was provided in **Figure 2** with a heatmap form, and the VOCs in the blank medium (artificial nectar medium) were excluded from the final results.

The composition of mVOCs was unique for each yeast strain. Among the 14 yeast strains, the control yeast *M. reukaufii* produced the highest number of mVOCs, while strain C31 produced the least number of mVOCs (only ethanol). Although the mVOC composition varied among the yeast strains, some common substances, such as ethanol, isoamyl alcohol (including 2-methyl-1-butanol), phenyl ethanol and 2,5-dimethyl furan, were produced by most yeasts. Ethanol was produced by all the tested yeasts under different temperatures. Twenty kinds of substances accounted for more than 1% of the mVOCs, and ethanol, 3-methyl-1-butanol, phenyl ethanol and 2-methyl-1-propanol were the major components (highest amount).

**Figure 3:**
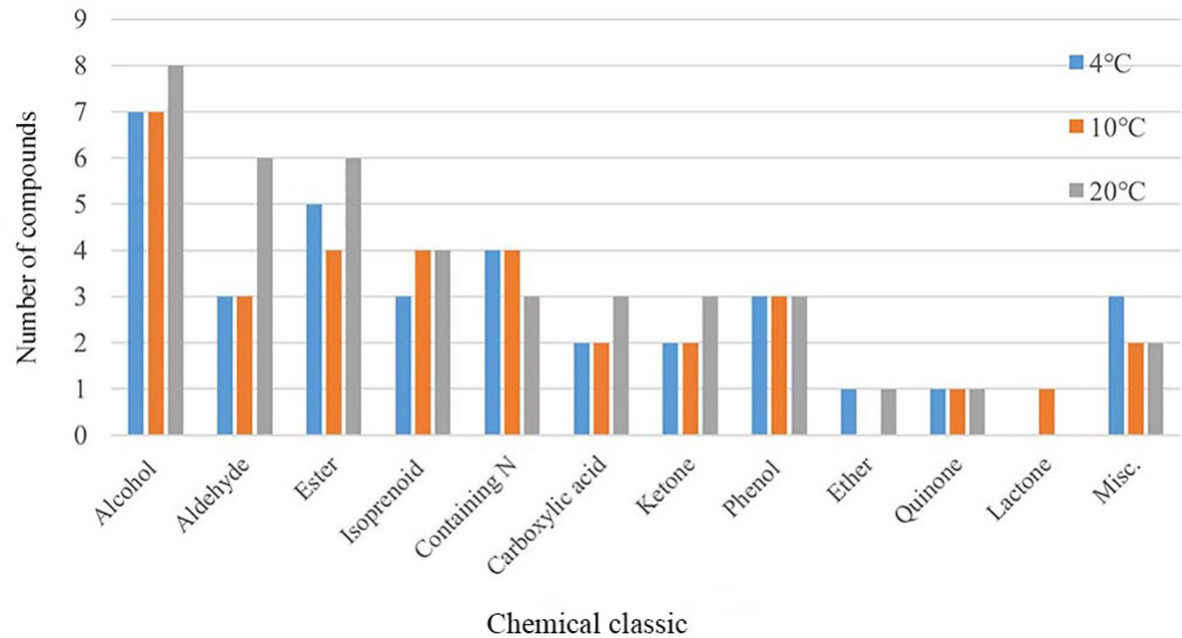
Chemical group distribution of VOCs produced by yeasts at varying temperatures.

To explore the effects of temperature on mVOCs production, all detected mVOCs were divided into different chemical categories, including alcohol, aldehyde, ketone, ester, lactone, isoprenoid, ether, carboxylic acid, phenol, quinone, nitrogenous compounds (containing N) and sulfur compounds (containing S). The substances that did not fall into any of above categories were grouped into ‘misc’. Among these groups, the most abundant groups were alcohol (8 compounds), ester (7 compounds) and aldehyde (6 compounds) (**Figure 3**). Chemicals containing alcohol, aldehyde and ester were most abundant at 20°C than at 4°C and 10°C; however, chemicals containing N were least abundant at 20°C. Lactone only existed at 10°C, and ester and ether were least abundant at 10°C compared with that at 4°C and 20°C. The misc group was more abundant at 4°C than at 10°C and 20°C, while isoprenoid was least abundant at 4°C. With an increase in temperature, the types of alcohols, aldehydes, esters, organic acids and ketones increased, while some low-temperature specific substances disappeared. In addition, the type of substances in the phenols and quinones group did not influenced by temperatures. Hence, incubation did influence the composition of mVOCs.

### 3.3 Antimicrobial effect of mVOCs *in vitro*

**Figure 4:**
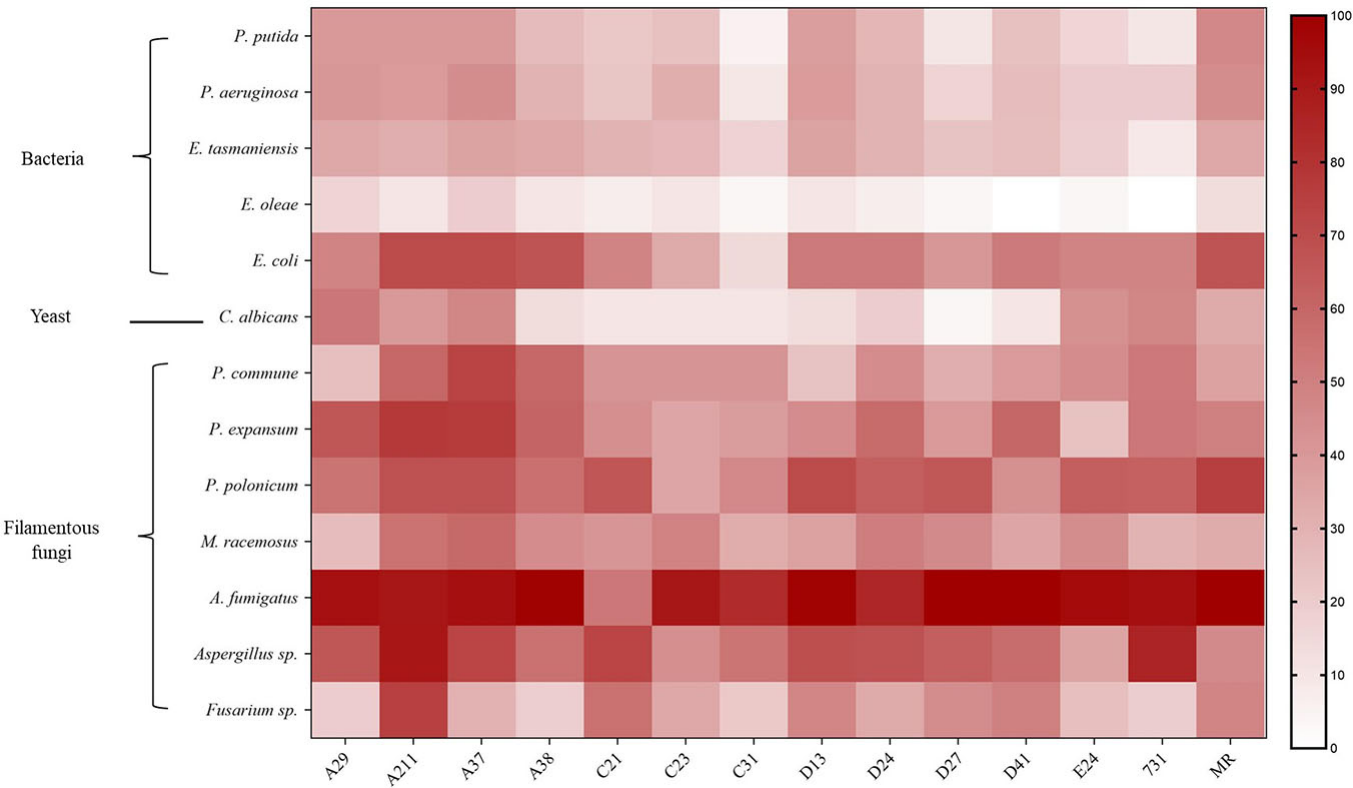
Inhibition rate of VOCs produced by yeasts on different microbes. X-axis indicates yeast strains; Y-axis indicates tested microbes; n=3; different color means different inhibition rate: the darker the color block was, the higher the inhibition rate was.

The results showed that mVOCs produced by the tested yeasts had an inhibitory effect on the growth of different microorganisms, and the inhibitory rate ranged from 3.33% to 100% (**Figure 4** and **Table S2**). The majority of the yeasts exhibited an inhibitory rate of more than 50%. The results showed that the growth of filamentous fungi were strongly inhibited (high inhibitory rates), while the growth of bacteria (especially *E. oleae*) were merely inhibited (low inhibitory rate). Overall, the mVOCs produced by strain A211 and strain A37 had the highest inhibitory effects, while the mVOCs produced by strain C31 and strain C21 had the weakest inhibitory effects. It was possibly caused by different sensitivities of the tested microorganisms to yeast mVOCs. For example, *A. fumigatus* was the most sensitive microbe, and mVOCs of all yeasts except that of strain C21 almost completely inhibited its growth. In contrast, *E. oleae* was the least sensitive microbe, and the inhibition rate with the majority of the mVOCs was less than 10%.

### 3.4 Effect of mVOCs on eed germination

**Figure 5:**
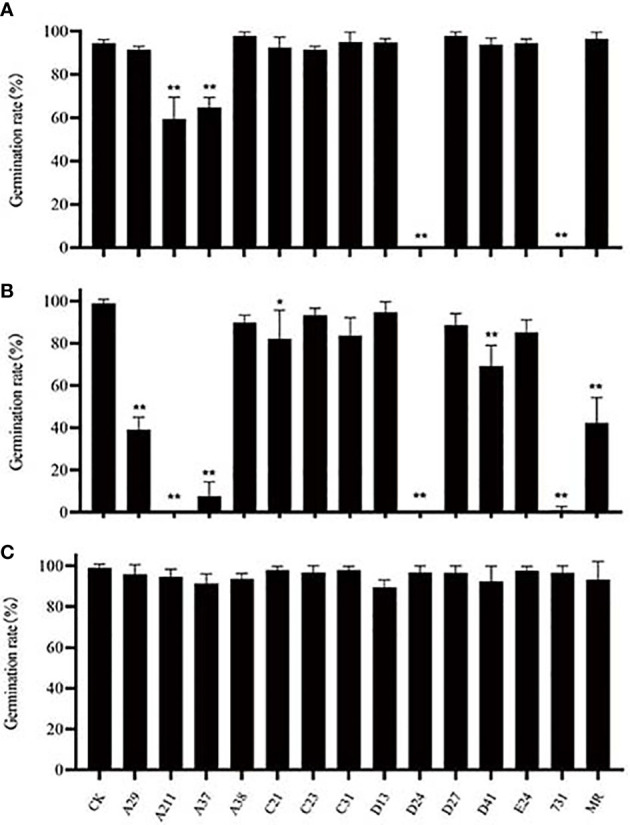
Effect of mVOCs on seed germination of *Arabidopsis thaliana* at varying incubating temperatures. **(A)** effect at 4°C; **(B)** effect at 10°C; **(C)** effect at 20°C. X-axis indicates yeast strains; Y-axis indicates seed germination rate; n=3; ‘*’ indicates P< 0.05, compared with water control at the same incubation temperature using Dunnett’s test; ‘**’ indicates P< 0.01, compared with water control at the same incubation temperature using Dunnett’s test. Standard error indicated in error bars.

The seeds of A. thaliana were used to explore the effect of yeast mVOCs on seed germination (**Figure 5**) while incubated at 20°C, 10°C and 4°C. It was found that when incubated at 20°C, the seed germination was unaffected, however, when incubated at lower temperature (10°C and 4°C), the seed germination rates of the yeast treatments (with yeast mVOCs) changed remarkably compared with control (**Figure 5**). While incubated at 4°C, the seed gemination was significantly inhibited by the mVOCs emitted by strain A37, A211, D24 and 731, compared with control. While incubated at 10°C, the seed gemination was significantly inhibited by the mVOCs emitted by strain A29, A37, A211, C21, D24, D41, 731 and *M. reukaufii*. It was also found that when the incubation temperature decreased, seed germination rates of the controls (without yeast mVOCs) was almost changeless, while that of the yeast treatments (with yeast mVOCs) changed remarkably (**Figure 5**). In general, seed germination rates at 4°C and 10°C were lower than that at 20°C, especially with the mVOCs of strain D24 and strain 731. Specifically, the germination rates at 10°C were lowest, indicating that mVOCs exerted a maximum inhibitory effect on seed germination at this temperature. Hence, some specific substances produced by yeasts at low temperatures (4°C and 10°C, especially 10°C) play an important role in inhibiting seed germination. Moreover, γ-valerolactone was produced by strain D41 only at 10°C and seed germination was more strongly inhibited (seed germination rate at 10°C was 20% lower than that at 4°C and 20°C) at this temperature, indicating that γ-valerolactone might have an inhibitory effect on seed germination.

The analysis of the results of the treatments with lower germination rates indicated that not all common components had inhibitory effects on seed germination. The inhibitory effects of mVOCs increased with an increase in the amount of benzene e thanol and a decrease in the amount of 2,5-dimethyl benzaldehyde, 2-octanone, β-cedrene, 2,4-di-tert-butylphenol and cedrol. Therefore, the inhibitory effect resulted not only because of the mVOC composition but also in the amount of substances. Certainly, the inhibition of seed germination might not be caused by a single component but also by the combination of several components.

### 3.5 Effect of mVOCs on A. thaliana growth

**Figure 6:**
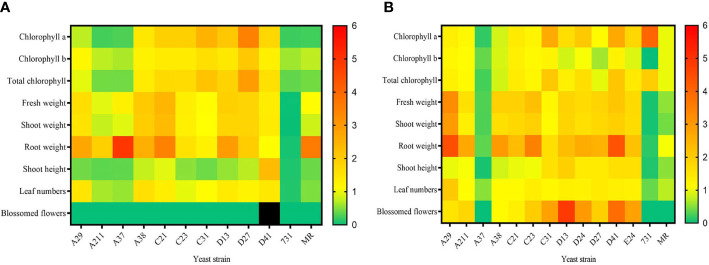
The effect of mVOCs produced by yeasts on plant growth at varying temperatures. **(A)** effect at 10°C; **(B)** effect at 20°C. X-axis indicates yeast strains; Y-axis indicates parameter folds compared to blank control; n=10; yellow indicates no difference (=1); green indicate inhibition (<1); orange and red indicate promotion (>1); black indicates strong promotion of plant growth (11.11 folds compared with control).

To explore the effect of mVOCs on plant growth and flowering, the isolated yeasts were co-cultured with *A. thaliana* at 10°C and 20°C. Detailed results of 20°C and 10°C were listed in **Tables S3** and **S4**, respectively. In general, the effect of yeast mVOCs on plant growth and flowering was basically uniform at 10°C and 20°C; however, the effect was higher in plants incubated at 20°C (**Figure 6**).

**Figure 6A** summarized the effect of yeast mVOCs on plants at 10°C. Overall, the mVOCs of all tested yeasts had a negative effect on plant flowering except for that strain D41. The mVOCs of strain D41 not only accelerated flowering but also induced plants to produce more flower buds (**Table S4**). Because flower buds would transform into flowers and seeds, it was concluded that mVOCs produced by strain D41 strongly promoted plant breeding. Furthermore, the mVOCs produced by all tested yeasts promoted root growth except for that of strain D41; however, the shoot height was enhanced only by the mVOCs of strain D41. Positive effects were also observed on chlorophyll synthesis, plant weight and leaf formation by a majority of the mVOCs of tested yeasts. Meanwhile, strain A37 and strain A211 inhibited chlorophyll synthesis, flower formation, leaf formation and plant elongation. The mVOCs of *M. reukaufii* (control yeast) had a totally negative effect on plant growth except for root growth.

As shown in **Figure 6B**, the mVOCs produced by most Arctic yeasts at 20°C had a positive effect on plant growth, with the exception of the mVOCs produced by strain A37 and strain 731. The most significant improvement was root formation and the root formation of the tested plants was 4.5 fold higher than that of the control. The mVOCs of yeasts made the plant more robust by increasing fresh weight, including shoot weight, total weight, and especially root weight. Regarding plant flowering at 20°C, the mVOCs of most yeast strains induced plants to produce more blossomed flowers compared with that of control; hence, the mVOCs accelerated plant flowering. Particularly, the mVOCs of strain D13 had an outstanding influence on the number of blossomed flowers, with a nearly 5-fold increase observed in the number of blossomed flowers compared with that of control.

The mVOCs of yeasts exerted different effects on plant growth and flowering at different incubation temperatures, indicating that the effect of mVOCs on plant growth was related to temperature. For example, the promoting effect on root growth of mVOCs of *M. reukaufii* (CCTCC SY2014008) at 10°C disappeared as the temperature rose to 20°C, and the positive effect of mVOCs produced by strain 731 on chlorophyll a and total chlorophyll enhanced at 20°C. In contrast, the mVOCs produced by strain A37 significantly promoted root weight (5 times that of control) at 20°C, whereas an inhibitory effect (0.35 times that of control) was observed at 10°C. It was speculated that the composition of mVOCs changed as the temperature fell; the change in mVOC composition maybe because of the adaptation of yeasts to non-optimal temperatures. However, the exact mechanism responsible for such changes at varying temperatures and its potential applications needs to be explored further.

### 3.6 Interaction between mVOCs and *A. thaliana*

While the co-culturing stage, the plant and yeast may interact, producing some special VOCs in the sealed space inside the yeast-plant co-culture system. The volatile metabolites detected in the co-cultured systems but not detected by sole yeasts or control were called ‘new VOC’. Total 17 types of new VOCs detected in this study were listed in **Table S5**. The new VOCs produced by the co-system of strain D13 and plant were the most diverse (11 types), while that of the co-system of strain 731 and plant were the least diverse (3 types). Two new VOCs (hendecane and tetradecane) were only detected in the co-system of strain D13-plant and strain D41-plant. The mVOCs of strain D13 and strain D41 had significantly positive effects on plant growth; therefore, it was speculated that hendecane and tetradecane played an important role in stimulating plant growth. Similarly, 1-hexanol and γ-muurolene were the new VOCs detected only in the co-system of strain 731 and plant; therefore, it was speculated that 1-hexanol and γ-muurolene inhibited plant growth. As a word, it was believed hendecane, tetradecane, 1-hexanol and γ-muurolene were special VOCs, and they may have some special influence to plant growth.

### 3.7 Effect of selected VOCs on *A. thaliana* growth

**Figure 7:**
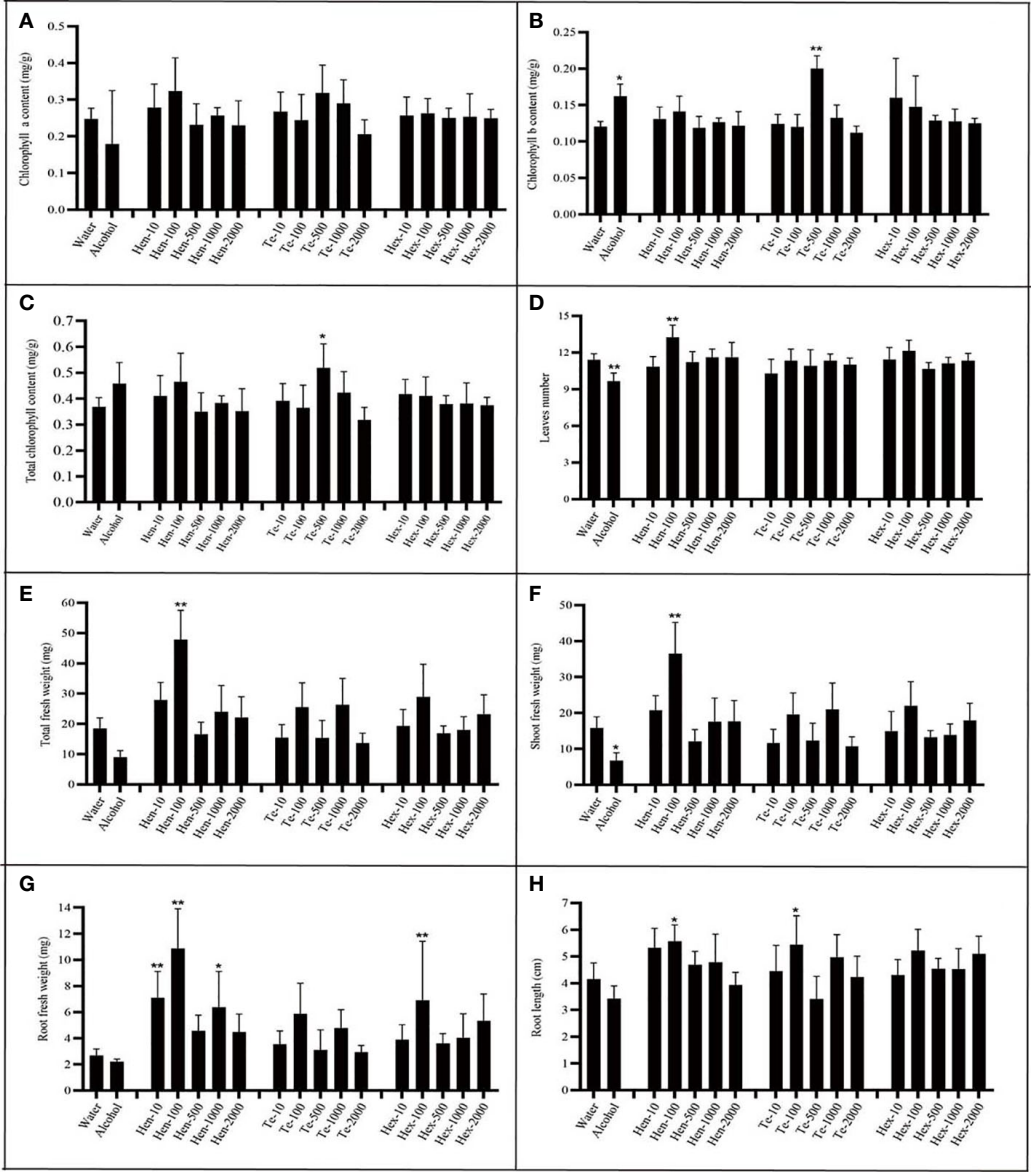
Effect of varying chemical concentrations on growth parameters. **(A)** chlorophyll a content; **(B)** chlorophyll b content; **(C)** total chlorophyll content; **(D)** leaf numbers; **(E)** total plant fresh weight; **(F)** shoot fresh weight; **(G)** root fresh weight; **(H)** largest root length. ‘Hen’ indicates hendecane; “Te” indicates tetradecane; ‘Hex’ indicates 1-hexanol; n=7; The number behind ‘-’ means the dose added (µg per plate). ‘*’ indicates P< 0.05, compared with water control using Dunnett’s test; ‘**’ indicates P< 0.01, compared with water control using Dunnett’s test. Standard error indicated in error bars.

To evaluate the effect of the new VOCs on plant growth, commercially available chemical standards were used. Because of the commercial non-availability of γ-muurolene, only the effects of hendecane, tetradecane and 1-hexanol were tested. Plants were incubated with varying doses of hendecane, tetradecane and 1-hexanol at 20°C, and the chlorophyll content, fresh weight, leaf numbers, flower numbers and longest root length of plants were measured and analyzed (**Figure 7** and **Figure S5**).

Analysis of results regarding hendecane indicated that only 100 µg dose could significantly stimulated plants to produce more leaves. Furthermore, at this dose, hendecane affected total plant fresh weight, shoot fresh weight, root fresh weight and root length. In other words, 100 µg hendecane significantly promoted plant growth as it had a positive effect on all tested parameters except for chlorophyll content. Both the 10 µg dose and 1000 µg of hendecane had a significantly positive effect on the root fresh weight but had no significant effect on root elongation compared with that of control. Overall, hendecane had a significantly positive effect on plant root growth (including root elongation and fresh weight increase), leaf emergence and fresh weight increase, but the degrees of this effect varied with the dose.

Analysis of results regarding tetradecane indicated that it could significantly increase the chlorophyll content (including total chlorophyll and chlorophyll b) of plants only at 500 µg dose. Furthermore, only 100 µg dose could significantly promote the root elongation of plant. However, The leaf numbers and the fresh weight (including total fresh weight, shoot fresh weight and root fresh weight) were all unaffected by tetradecane at all tested doses. As for 1-hexanol, only at the dose of 100 µg, root fresh weight could be significantly promoted at all growth parameters and at all tested doses.

The hypothesized promoting or inhibiting effect of these three chemicals did not exactly confirm with the experimental results. Hendecane, tetradecane and 1-hexanol all had promoting effects on plant growth, while the degrees and aspects of these effects differed at different doses. The results indicate that a complicated mechanism may be responsible for the effects of VOCs on plant growth at different doses.

## 4 Discussion

Microorganisms in flowers may play an important role in the Arctic ecosystem through the VOCs they produced. Among the studied Arctic plants, *Silene acaulis*, *Saxifraga oppositifolia* and *Saxifraga cespitosa* has the ability to synthesize and secrete nectar (Swales, 1979). Nectar is a high-concentration sugar solution mainly composed of glucose, fructose and sucrose. The Yeasts isolated from the flowers of the five Arctic plant species were tolerant to high concentrations of sucrose, fructose and glucose, indicating their possible presence in nectar. Currently, the reported nectar yeasts include the members of basidiomycota and ascomycota, such as Metschnikowia family (typically, *M. reukaufii*), *Candida* sp., *Rhodotorula* sp., *Sporobolomyces* sp. and *Cryptococcus* sp. (Zhang et al., 2017). The 13 isolated yeasts from Arctic flowers not only include *R. mucilaginosa* and *Cryptococcus* sp. that reported to exist in the nectar of non-Arctic plants (Zhou et al., 2013; Pozo et al., 2014; Ma et al., 2018), *Cystobasidium laryngis*, *Mrakia gelida*, *Vishniacozyma victoriae*, *Debaryomyces hansenii*, *Goffeauzyma gilvescens*, *Cystofilobasidium* sp., *Microbotryum* sp. and *Sporidiobolus* sp. that reported to exist in non-Arctic plants (Zhou, 2020a; Zhou, 2020b), but also include *Tausonia pullulans*, *Microbotryum silenes-acaulis* and *Sporidiobolus salmonicolor* that isolated from flowers for the first time. These yeasts produced different mVOCs, and the mVOCs of the same genus are not always the same. In this research, the component of mVOCs of yeasts A211 and A37 belonging to *Cystofilobasidium* were highly similar; however, the component of mVOCs of yeasts D24 and 731 belonging to *Mrakia* were significantly different.

Previous studies have demonstrated that temperature has a significant effect on mVOC synthesis (Polizzi et al., 2012; Misztal et al., 2018), including mVOC composition and quantity. Because of global warming, the temperature in the Arctic region has been increasing steadily (IPCC, 2013), which may affect the effects of Arctic mVOCs on plants and insects. In this study, some mVOCs appeared with a rise in temperature, while some low-temperature specific mVOCs disappeared. As the temperature rose, (-)-cubenol, 2,4-pentadienal, octanal, 1-nonanal, 6,6-dimethyl tetrahydro-2H-pyran-2-one, isobutyric acid and ethyl phenylacetate were detected, while pyridine and 2-acetyl furan disappeared. Previous studies have reported that these substances affect microbial growth, insect behavior, etc. For example, insects had strong electro-antennogram (EAG) responses to octanal, 1-nonanal and (-)-cubenol (Lago et al., 2006; Rebora et al., 2012; Xiang et al., 2017). Insects acutely recognize and sense these substances, thereby affecting their further behavior. Because (-)-cubenol is a plant-origin volatile metabolite, it was speculated that yeasts enhanced their competitiveness by synthesizing and releasing volatile metabolites that mimic plant metabolites. Octanal inhibits spore germination of filamentous fungi by destroying membrane integrity (Dou et al., 2017), and this might be a niche competitive strategy of yeasts. A previous study reported pyridine to be neurotoxic (Lin et al., 2020), likely affecting the nervous system of insects. Therefore, these mVOCs may affect the growth of plants and the behavior of insects in the Arctic region, thereby further affecting the Arctic ecosystem. This study only focused on the effect of yeast mVOCs on plants, and the future plan is to explore their effect on insect behavior. For this, it would be better if field experiments are conducted in the Arctic region.

The results of the yeast and *A. thaliana* co-culture system indicated that although the mVOCs of the Arctic yeasts generally had a stronger promoting effect on plant growth at 20°C, the strongest promoting effect on root growth (strain A37), chlorophyll synthesis (strain D27), and plant flowering (strain D41) was observed at 10°C. To the best of our knowledge, this is the first time a study has reported that yeast mVOCs effect on plant growth is temperature-related. It was speculated that increased temperature improved the growth-promoting effect of most yeast mVOCs, while the growth-promoting effect of some yeast mVOCs decreased with increasing temperature. Further experiments should be performed by inoculating yeasts into the nectar of Arctic flowers, thereby increasing the understanding of the possible effects of climate change on Arctic plants, insects, and the ecosystem. Through the detection of volatiles in the co-culture system of plant and yeast, it was observed that yeast and plant interact to produce some new VOCs; these VOCs may be produced either by plants or by yeast and mainly included alkanes and alcohols. Co-culturing experiments of plants and single pure chemicals demonstrated that hendecane, tetradecane and 1-hexanol promoted plant growth at specific concentrations; these results were consistent with that of previous studies (Blom et al., 2011; Jishma et al., 2017).

In addition, mVOCs of Arctic yeasts exhibited good inhibitory effects on the growth of bacteria, yeast and filamentous fungi, especially on filamentous fungi. Interestingly, the mVOCs of Arctic yeasts had a stronger inhibitory effect on the strains of *Penicillium* isolated from the Arctic than the non-Arctic strain of *Penicillium*; therefore, it was speculated that this might be a strategy of Arctic yeasts to improve its ecological niche. A previous study demonstrated that sesquiterpenes have a strong inhibitory effect on fungal growth (Brasch et al., 2014), and octanal inhibits spore germination of filamentous fungi by destroying its membrane integrity (Dou et al., 2017). Hence, these volatile substances might play an important role in inhibiting fungal growth, and experiments are required in the future to verify their real role. Furthermore, previous studies have indicated the potential applications of mVOCs in the fields of food, medical, agriculture and ecology (Insam and Seewald, 2010; Lemfack et al., 2018; Contarino et al., 2019; Fang et al., 2020; Guevara-Avendaño et al., 2020; Poveda, 2021); however, further research is required to confirm this.

## 5 Conclusions

mVOCs emitted by 13 yeast species isolated from Arctic flowers were detected at varying incubation temperatures, and results indicated that the composition and content of mVOCs were significantly influenced by temperature. Through co-culturing experiments of plants and yeasts, it was observed that yeast mVOCs promoted the growth and flowering of plants, while inhibited seed germination. Although the mVOCs of Arctic yeasts generally had stronger promoting effect on plant growth at 20°C compared with that at 10°C, the strongest promoting effect of yeast mVOCs on root growth, flowering and chlorophyll synthesis was observed at 10°C. When yeasts were co-cultured with plants, some new VOCs were produced; among these, 10 µg hendecane, 100 µg hendecane, 100 µg tetradecane, and 100 µg 1-hexanol significantly promoted fresh weight and root growth of *A. thaliana*. These results suggested that increasing Arctic temperatures may alter plant growth by altering microbial VOCs. This study not only laid a foundation for exploring the interactions between Arctic microorganisms and plants and their possible responses to temperature changes but also demonstrated the potential application of mVOCs emitted by Arctic yeasts in promoting plant growth and inhibiting microbial growth.

## Data availability statement

The original contributions presented in the study are publicly available. This data can be found here: https://www.ncbi.nlm.nih.gov/;

Tausonia pullulans A29 OQ121660

Cystofilobasidium capitatum A37 OQ121664

Debaryomyces hansenii A38 OQ121666

Cystofilobasidium sp. A211 OQ121667

Vishniacozyma victoriae C21 OQ121668

Rhodotorula mucilaginosa C23 OQ121669

Microbotryum silenes-acaulis C31 OQ121670

Goffeauzyma gilvescens D13 OQ121671

Mrakia gelida D24 OQ121672

Cystobasidium laryngis E24 OQ121673

Sporidiobolus salmonicolor D27 OQ121674

Cryptococcus sp. D41 OQ121675

Mrakia blollopis 731 OQ121676

Metschnikowia reukaufii MR. OQ121677.

## Author contributions

FP conceived, designed the project, and provided constructive suggestions on the first draft. YY collected field samples. JN performed lab experiments and statistical analyses, and wrote the manuscript. XL and SZ provided constructive foundation on this study. YZ, YL, XP, and JH made contribution to experiments. All authors read and approved the final manuscript.
