# Supplementary material for: Identification and functional studies of microbial volatile organic compounds produced by Arctic flower yeasts
Source: Front Plant Sci. 2023 Jan 5;13:941929. doi: 10.3389/fpls.2022.941929 (PMC9850290; doi:10.3389/fpls.2022.941929)
Supplement: Supplementary file 1 [file DataSheet_1.docx]

Supplementary Material

# Supplementary Tables

**Table S1.** Information of the tested microorganisms.

| **Category** | **Species** | **Preservation number** | **Culture medium** |
| --- | --- | --- | --- |
| Bacteria | *Pseudomonas putida* | CCTCC AB2014017 | NA |
|  | *Pseudomonas aeruginosa* | CCTCC AB93066 | NA |
|  | *Erwinia oleae* | CCTCC AB2014255 | NA |
|  | *Erwinia tasmaniensis* | CCTCC AB2014256 | NA |
|  | *Escherichia coli* | CCTCC AB204033 | LB |
| Yeast | *Candida albicans* | CCTCC AY2022003 | LB |
| Filamentous fungi | *Penicillium commune* | CCTCC AF93302 | PDA |
|  | *Penicillium expansum* | CCTCC S2022025 | PDA |
|  | *Penicillium polonicum* | CCTCC S2022026 | PDA |
|  | *Mucor racemosus* | CCTCC AF93267 | PDA |
|  | *Aspergillus fumigatus* | CCTCC AF93048 | PDA |
|  | *Aspergillus sp.* | CCTCC AF2019015 | PDA |
|  | *Fusarium sp.* | CCTCC AF93230 | PDA |
|  | *Trichoderma sp.* | CCTCC AF2018015 | PDA |

Note: CCTCC (China Center for Type Culture Collection; Wuhan, Hubei, China); NA, Nutrient agar.

**Table S2.** Inhibition rates of yeast VOCs to microbial growth.

| **Yeast**  **strain** | **Inhibition rate (%, Mean±SD)** | | | | | | | | |
| --- | --- | --- | --- | --- | --- | --- | --- | --- | --- |
|  | ***E. coli*** | ***C. albicans*** | ***P. commune*** | ***P. expansum*** | ***P. polonicum*** | ***Aspergillus sp.*** | ***A. fumigatus*** | ***M. racemosus*** | ***Fusarium sp.*** |
| A29 | 48.15±12.83^b^ | 53.33±5.77^d^ | 25.00±6.61^a^ | 65.69±2.12^a^ | 54.36±7.48^a^ | 65.71±33.52^a^ | 93.52±11.23^b^ | 26.23±13.98^a^ | 20.00±17.33^a^ |
| A211 | 70.37±6.42^b^ | 40.00±0.00^c^ | 59.17±13.77^a^ | 77.33±19.66^b^ | 68.19±25.77^a^ | 91.23±11.05^b^ | 90.97±12.77^b^ | 54.64±7.35^b^ | 74.67±19.91^b^ |
| A37 | 70.37±6.42^b^ | 46.67±5.77^d^ | 73.33±5.20^b^ | 76.72±20.16^b^ | 67.50±5.22^a^ | 72.89±22.23^a^ | 93.98±10.42^b^ | 58.47±7.35^b^ | 30.22±10.01^a^ |
| A38 | 66.67±11.11^b^ | 13.33±5.77^a^ | 59.17±12.33^a^ | 60.78±7.65^a^ | 55.74±3.17^a^ | 55.34±5.52^a^ | 99.54±0.80^b^ | 45.08±15.36^a^ | 19.11±6.01^a^ |
| C21 | 48.15±6.42^b^ | 10.00±0.00^a^ | 41.67±22.41^a^ | 43.63±9.43^a^ | 66.11±17.89^a^ | 72.89±23.48^a^ | 52.78±5.89^a^ | 41.26±9.57^a^ | 55.11±19.79^a^ |
| C23 | 33.33±11.11^a^ | 10.00±0.00^a^ | 41.67±13.77^a^ | 35.05±10.45^a^ | 34.99±4.32^a^ | 43.78±11.84^a^ | 90.74±8.14^b^ | 48.36±10.66^a^ | 33.78±22.36^a^ |
| C31 | 14.81±6.42^a^ | 10.00±0.00^a^ | 41.67±6.29^a^ | 38.11±2.81^a^ | 46.06±2.07^a^ | 53.75±1.38^a^ | 82.41±16.22^b^ | 32.51±3.10^a^ | 20.89±7.81^a^ |
| D13 | 51.85±6.42^b^ | 13.33±5.77^a^ | 23.33±1.44^a^ | 44.85±17.54^a^ | 70.26±23.32^a^ | 68.90±25.20^a^ | 98.61±2.41^b^ | 36.34±4.21^a^ | 47.11±18.10^a^ |
| D24 | 51.85±6.42^b^ | 20.00±0.00^b^ | 45.00±13.23^a^ | 57.72±5.51^a^ | 62.66±14.52^a^ | 68.10±1.38^a^ | 84.72±18.16^b^ | 50.55±8.69^a^ | 33.33±6.11^a^ |
| D27 | 40.74±6.42^a^ | 3.33±0.00^a^ | 31.67±2.89^a^ | 39.34±9.55^a^ | 65.42±29.14^a^ | 62.52±9.96^a^ | 100.00±0.00^b^ | 45.63±8.05^a^ | 44.44±2.04^a^ |
| D41 | 51.85±6.42^b^ | 10.00±0.00^a^ | 39.17±19.42^a^ | 59.56±7.35^a^ | 43.29±7.85^a^ | 56.94±35.24^a^ | 100.00±0.00^b^ | 34.97±10.38^a^ | 49.78±29.70^a^ |
| E24 | 48.15±12.83^b^ | 43.33±5.77^c^ | 45.00±20.46^a^ | 24.02±1.06^a^ | 62.66±14.52^a^ | 35.41±7.18^a^ | 95.14±6.87^b^ | 44.54±0.47^a^ | 24.89±15.91^a^ |
| 731 | 48.15±6.42^b^ | 46.67±5.77^d^ | 52.50±5.00^a^ | 52.82±14.04^a^ | 61.96±9.58^a^ | 85.65±6.33^a^ | 93.75±8.84^b^ | 29.51±3.57^a^ | 19.33±6.60^a^ |
| MR | 66.67±11.11^b^ | 33.33±5.77^c^ | 36.67±35.91^a^ | 49.75±14.97^a^ | 75.10±17.97^a^ | 45.77±7.31^a^ | 100.00±0.00^b^ | 32.79±7.51^a^ | 47.56±33.32^a^ |

Note: Different lowercase letters indicate significant differences within a column according to Tukey-Kramer test (p < 0.05).

**Table S3.** Influence of VOC of yeasts to the growth of *A. thaliana* at 20℃.

|  | **Yeast strain** | **Chlorophyll content (mg/g)** | | | **Fresh weight (mg)** | | | **Shoot height**  **(cm)** | **Leaf numbers** | **Blossomed**  **flowers** |
| --- | --- | --- | --- | --- | --- | --- | --- | --- | --- | --- |
|  |  | **Chlorophyll**  **a**  **content** | **Chlorophyll**  **b**  **content** | **Total**  **chlorophyll**  **content** | **Total**  **fresh**  **weight** | **Root weight** | **Shoot weight** |  |  |  |
| Experiment  1 | CK | 0.16±0.04 | 0.07±0.01 | 0.22±0.06 | 111.00±63.14 | 22.83±15.43 | 88.17±48.38 | 7.15±1.23 | 18.50±2.88 | 1.83±0.75 |
|  | A29 | 0.19±0.10 | 0.07±0.03 | 0.26±0.15 | 361.75±88.83** | 102.00±32.07** | 259.75±59.30** | 6.53±0.74 | 37.75±10.31** | 2.75±1.50 |
|  | A211 | 0.12±0.06 | 0.05±0.02 | 0.17±0.08 | 176.25±40.34 | 61.75±20.19 | 114.50±37.55 | 6.89±0.80 | 19.00±2.16 | 3.25±1.26 |
|  | A38 | 0.16±0.08 | 0.07±0.02 | 0.23±0.12 | 209.75±84.79 | 67.25±36.93 | 142.50±52.11 | 5.83±0.75 | 20.25±1.50 | 2.00±0.82 |
|  | C21 | 0.20±0.05 | 0.08±0.02 | 0.28±0.08 | 206.80±54.15 | 52.80±29.18 | 154.00±56.47 | 6.51±1.17 | 20.20±2.28 | 2.40±2.30 |
|  | C23 | 0.18±0.04 | 0.07±0.01 | 0.24±0.06 | 247.00±88.19* | 79.80±46.69* | 167.20±46.59* | 6.74±1.02 | 24.60±2.88 | 3.60±2.88 |
|  | MR | 0.14±0.02 | 0.06±0.01 | 0.19±0.03 | 60.80±13.85 | 22.20±8.93 | 38.60±14.72 | 3.82±1.28** | 13.40±2.41 | 0.00±0.00 |
| Experiment  2 | CK | 0.10±0.02 | 0.07±0.02 | 0.17±0.02 | 141.00±70.00 | 12.58±8.78 | 128.58±63.17 | 4.11±1.39 | 25.25±7.35 | 1.75±1.54 |
|  | A37 | 0.02±0.01 | 0.03±0.01** | 0.04±0.02 | 41.00±17.88** | 4.40±3.47 | 36.40±15.85** | 0.31±0.09** | 13.30±2.67** | 0.00±0.00 |
|  | C31 | 0.27±0.10* | 0.08±0.03 | 0.35±0.15** | 161.00±47.43 | 20.27±9.14 | 139.82±41.70 | 6.50±1.30** | 28.91±7.23 | 4.91±1.92** |
|  | D13 | 0.17±0.03 | 0.05±0.01 | 0.22±0.05 | 261.00±70.84** | 28.64±7.03* | 232.36±65.07** | 7.85±2.15** | 32.55±6.89* | 8.55±3.62** |
|  | D24 | 0.21±0.03 | 0.07±0.01 | 0.27±0.05 | 239.55±77.77** | 33.18±14.23** | 206.36±65.70** | 5.75±1.06* | 31.27±7.10 | 5.18±1.08** |
|  | D27 | 0.11±0.05 | 0.04±0.01 | 0.15±0.06 | 258.08±48.80** | 31.25±10.62** | 226.83±40.55** | 5.79±1.53* | 34.75±7.29** | 3.33±0.98 |
|  | D41 | 0.27±0.04* | 0.08±0.01 | 0.36±0.06** | 296.17±88.03** | 55.00±24.46** | 244.50±70.11** | 6.57±1.97** | 32.92±6.73* | 6.75±2.96** |
|  | E24 | 0.18±0.08 | 0.06±0.02 | 0.24±0.11 | 241.73±48.59** | 27.45±6.27* | 214.36±46.64** | 6.59±1.13** | 32.09±4.85 | 4.91±1.64** |
|  | 731 | 0.40±0.17** | 0.00±0.00** | 0.33±0.14** | 10.25±7.76** | 1.88±2.10 | 8.38±5.76** | 0.43±0.29** | 9.75±1.16** | 0.00±0.00 |

Note: The test was carried out in two times, and the control (CK) was set twice; '*' indicates P < 0.05, compared with control using Dunnett’s test; '**' indicates P < 0.01, compared with control using Dunnett’s test.

**Table S4.** Influence of VOC of yeasts to the growth of *A. thaliana* at 10℃.

| **Yeast strain** | **Chlorophyll content (mg/g)** | | | **Fresh weight (mg)** | | | **Shoot height**  **(cm)** | **Leaf numbers** | **Blossomed**  **flowers** |  |
| --- | --- | --- | --- | --- | --- | --- | --- | --- | --- | --- |
|  | **Chlorophyll**  **a**  **content** | **Chlorophyll**  **b**  **content** | **Total**  **chlorophyll**  **content** | **Total**  **fresh**  **weight** | **Root weight** | **Shoot weight** |  |  |  | **Unblossomed**  **flowers** |
| Control | 0.06±0.01 | 0.04±0.00 | 0.10±0.01 | 331.00±128.83 | 33.30±26.00 | 297.67±108.18 | 3.21±1.49 | 35.67±4.61 | 0.11±0.33 | 7.78±6.96 |
| A29 | 0.05±0.01 | 0.04±0.02 | 0.09±0.03 | 544.50±131.44** | 79.67±76.11* | 454.88±97.74** | 1.36±0.40** | 53.25±8.40** | 0.00±0.00 | 0.00±0.00 |
| A211 | 0.02±0.00 | 0.03±0.00 | 0.04±0.01 | 292.10±85.93 | 63.70±46.50 | 228.40±48.69 | 1.13±0.13** | 23.00±4.29** | 0.00±0.00 | 0.00±0.00 |
| A37 | 0.02±0.00 | 0.03±0.00 | 0.04±0.01 | 423.60±69.48 | 163.00±42.52** | 260.60±37.99 | 1.17±0.12** | 20.70±3.20** | 0.00±0.00 | 0.00±0.00 |
| A38 | 0.09±0.02 | 0.05±0.00 | 0.14±0.03 | 673.11±138.32** | 84.56±27.68* | 588.56±120.47** | 2.46±1.04 | 56.78±9.58** | 0.00±0.00 | 6.22±11.72 |
| C21 | 0.12±0.03 | 0.05±0.01 | 0.17±0.04 | 800.11±80.04** | 114.11±34.56** | 686.00±95.07** | 2.83±0.57 | 47.56±12.19** | 0.00±0.00 | 0.00±0.00 |
| C23 | 0.12±0.05 | 0.05±0.01 | 0.17±0.07 | 437.67±87.18 | 51.56±16.82 | 386.11±82.26 | 1.71±0.27** | 32.11±5.13 | 0.00±0.00 | 0.00±0.00 |
| C31 | 0.15±0.06** | 0.06±0.02* | 0.22±0.08** | 338.33±47.77 | 41.44±13.50 | 296.89±49.69 | 1.34±0.38** | 37.33±7.14 | 0.00±0.00 | 0.00±0.00 |
| D13 | 0.13±0.03* | 0.06±0.01 | 0.19±0.04* | 639.22±128.35** | 96.78±32.58** | 542.44±101.86** | 1.83±0.21** | 50.33±12.05** | 0.00±0.00 | 0.56±1.67 |
| D27 | 0.21±0.06** | 0.08±0.02** | 0.30±0.09** | 609.78±69.14** | 66.00±25.10 | 543.78±48.18** | 2.30±0.41 | 43.11±7.88 | 0.00±0.00 | 1.89±3.95 |
| D41 | 0.11±0.01 | 0.05±0.00 | 0.16±0.01 | 445.67±102.87 | 33.22±15.09 | 412.44±95.50** | 7.37±1.60** | 51.44±7.47** | 1.22±1.64 | 26.33±11.00** |
| 731 | 0.01±0.00 | 0.02±0.00 | 0.04±0.00 | 6.00±1.41** | 2.11±0.78 | 3.89±1.27** | 0.31±0.03** | 3.89±0.78** | 0.00±0.00 | 0.00±0.00 |
| MR | 0.01±0.00 | 0.03±0.00 | 0.04±0.01 | 354.90±58.91 | 118.90±48.78** | 236.00±23.53 | 1.31±0.09** | 17.00±1.94** | 0.00±0.00 | 0.00±0.00 |
| D24 | ND | ND | ND | ND | ND | ND | ND | ND | ND | ND |
| E24 | - | - | - | - | - | - | - | - | - | - |

Note: “ND” means the plant was too small to measure; “-” means data was lacked due to contamination; '*' indicates P < 0.05, compared with control using Dunnett’s test; '**' indicates P < 0.01, compared with control using Dunnett’s test.

**Table S5.** Detected new VOCs in yeast- *A. thaliana* co-culture system.

|  | **New VOC** | | | | | | | | **Peak Area (unit, ×10^6^)，Mean±SD** | | |
| --- | --- | --- | --- | --- | --- | --- | --- | --- | --- | --- | --- |
| **Classic** | | | **Name** | **CAS number** | | **RI**  **(min)** | **Chemical formula** | **MW** | **Strain**  **731** | **Strain**  **D13** | **Strain**  **D41** |
| Alcohol | | 1-Hexanol | | | 111-27-3 | 7.738 | C_6_H_14_O | 102 | 298.1±14.7 | - | - |
|  | | 2-Hexyl-1-octanol | | | 19780-79-1 | 15.665 | C_14_H_30_O | 214 | - | 2.5±0.1 | - |
| Organic acid | | Isobutyric acid | | | 79-31-2 | 5.572 | C_4_H_8_O_2_ | 88 | - | - | 18.1±10.1 |
| Alkane | | 2-Methyl butane | | | 78-78-4 | 10.152 | C_5_H_12_ | 72 | - | 7.2±0.1 | - |
|  | | Decane | | | 124-18-5 | 12.506 | C_10_H_22_ | 142 | - | - | 1.6±0.2 |
|  | | Hendecane | | | 1120-21-4 | 12.602 | C_11_H_24_ | 156 | - | 1.4±0.0 | 1.7±0.4 |
|  | | 2,6-Dimethyl undecane | | | 17301-23-4 | 14.354 | C_13_H_28_ | 184 | - | 2.2±0.1 | - |
|  | | 2,3,4-Trimethyl decane | | | 62238-15-7 | 14.490 | C_13_H_28_ | 184 | - | 3.8±0.1 | - |
|  | | n-Tridecane | | | 629-50-5 | 14.951 | C_13_H_28_ | 184 | 1.4±0.5 | 15.4±0.2 | - |
|  | | Tetradecane | | | 629-59-4 | 15.269 | C_14_H_30_ | 198 | - | 2.1±0.3 | 6.8±0.8 |
|  | | 2,6,10-Trimethyl tetradecane | | | 14905-56-7 | 14.166 | C_17_H_36_ | 240 | - | 3.1±0.3 | - |
|  | | n-Heptadecane | | | 629-78-7 | 15.399 | C_17_H_36_ | 240 | - | 11.4±5.0 | - |
|  | | Octadecane | | | 593-45-3 | 18.114 | C_18_H_38_ | 254 | - | 3.6±0.4 | - |
|  | | 2,6-Dimethyl heptadecane | | | 54105-67-8 | 14.951 | C_19_H_40_ | 268 | - | 3.5±0.5 | - |
| Terpene | | (+)-Aromadendrene | | | 489-39-4 | 17.464 | C_15_H_24_ | 204 | - | - | 2.2±1.3 |
|  | | γ-Muurolene | | | 30021-74-0 | 17.641 | C_15_H_24_ | 204 | 15.6±13.6 | - | - |
|  | | (-)-Thujopsen | | | 470-40-6 | 17.771 | C_15_H_24_ | 204 | - | - | 1.2±0.1 |

Note: MW, molecular weight; RI, Retention time; -, not detected.

# Supplementary Figures


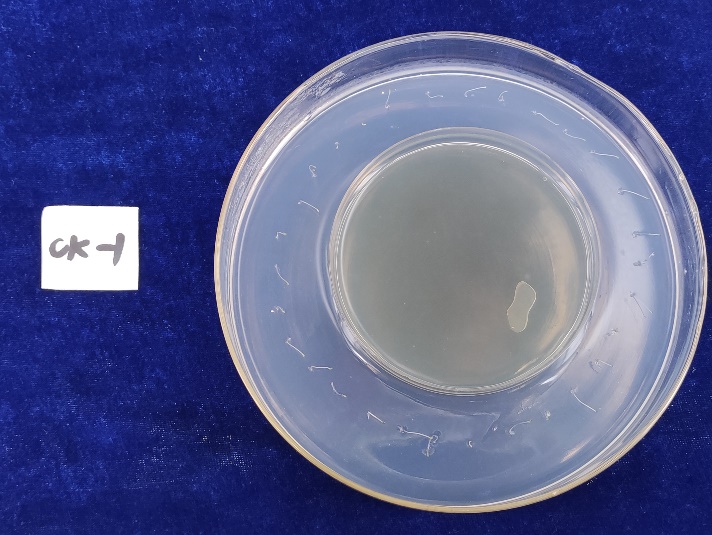


Artificial nectar and yeast

Plant culture medium

Seed of *A. thaliana*

**Figure S1.** Inside space in the experiment of the effect of mVOCs on seed germination.


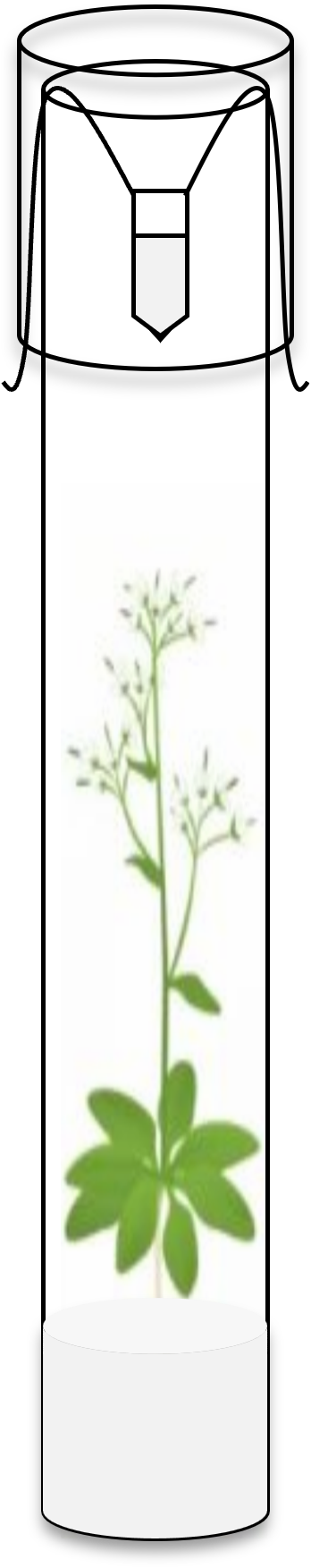


Artificial nectar and yeast

*A. thaliana*

Plant culture medium

Lid-moved EP tube (1.5 mL)

**Figure S2.** Mode pattern of mVOC- *A. thaliana* co-culture system.


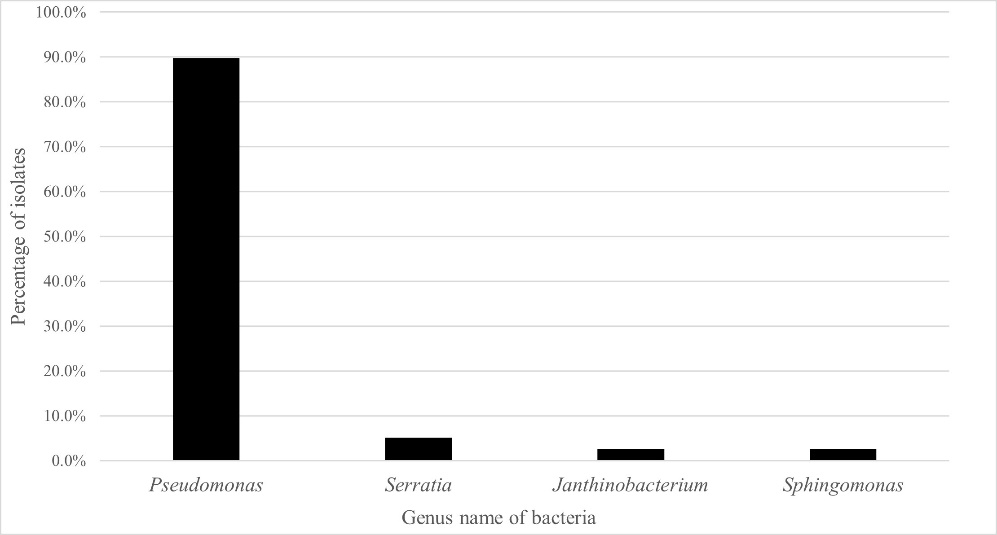


**Figure S3.** Genera distribution of all isolated yeasts from Arctic flowers.


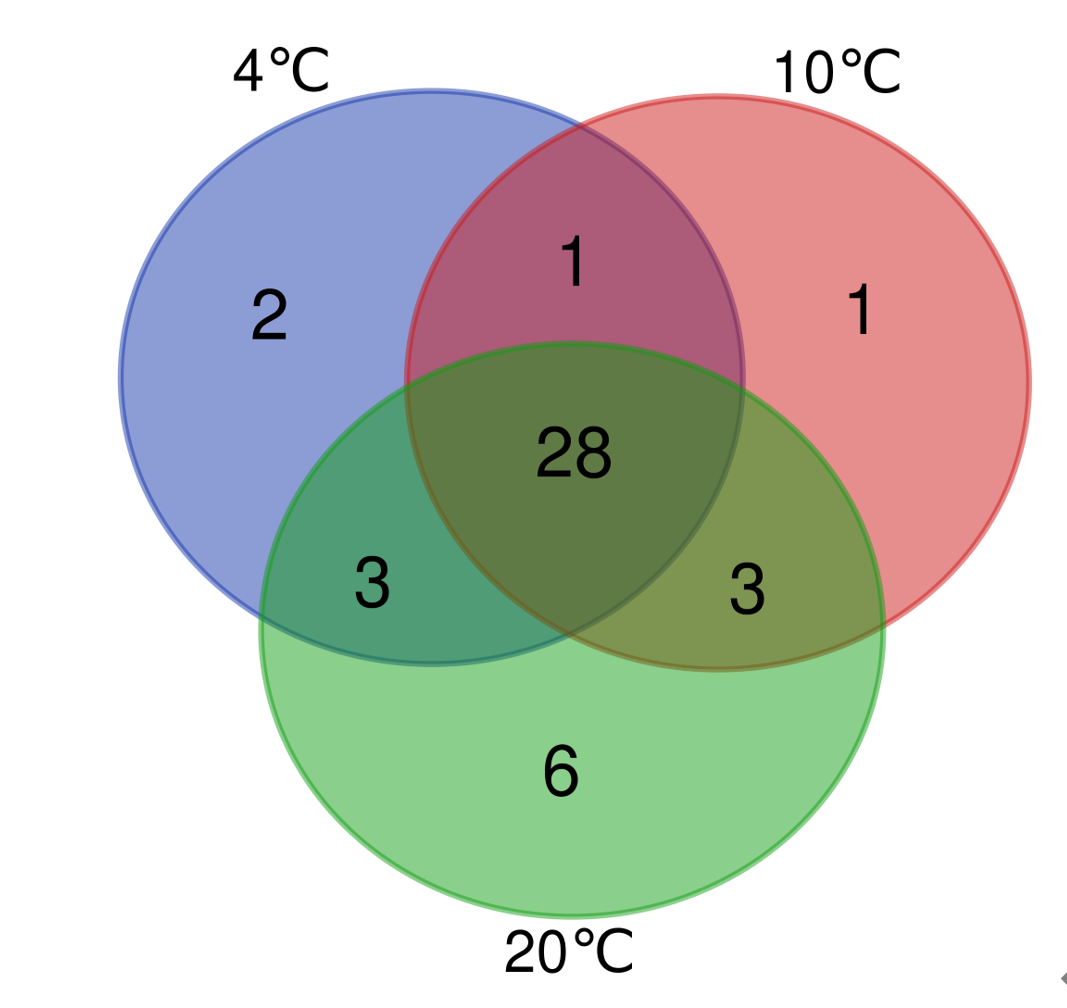


**Figure S4.** Venn diagram of the mVOC components at varying incubation temperatures.


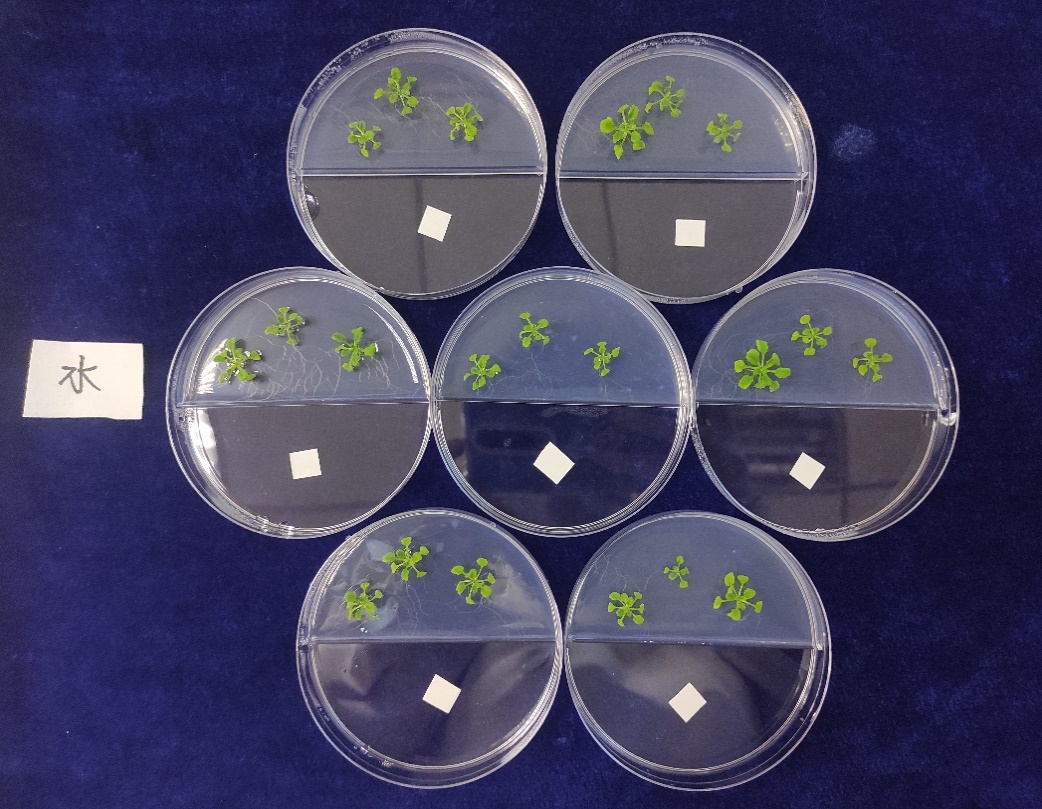


**a**


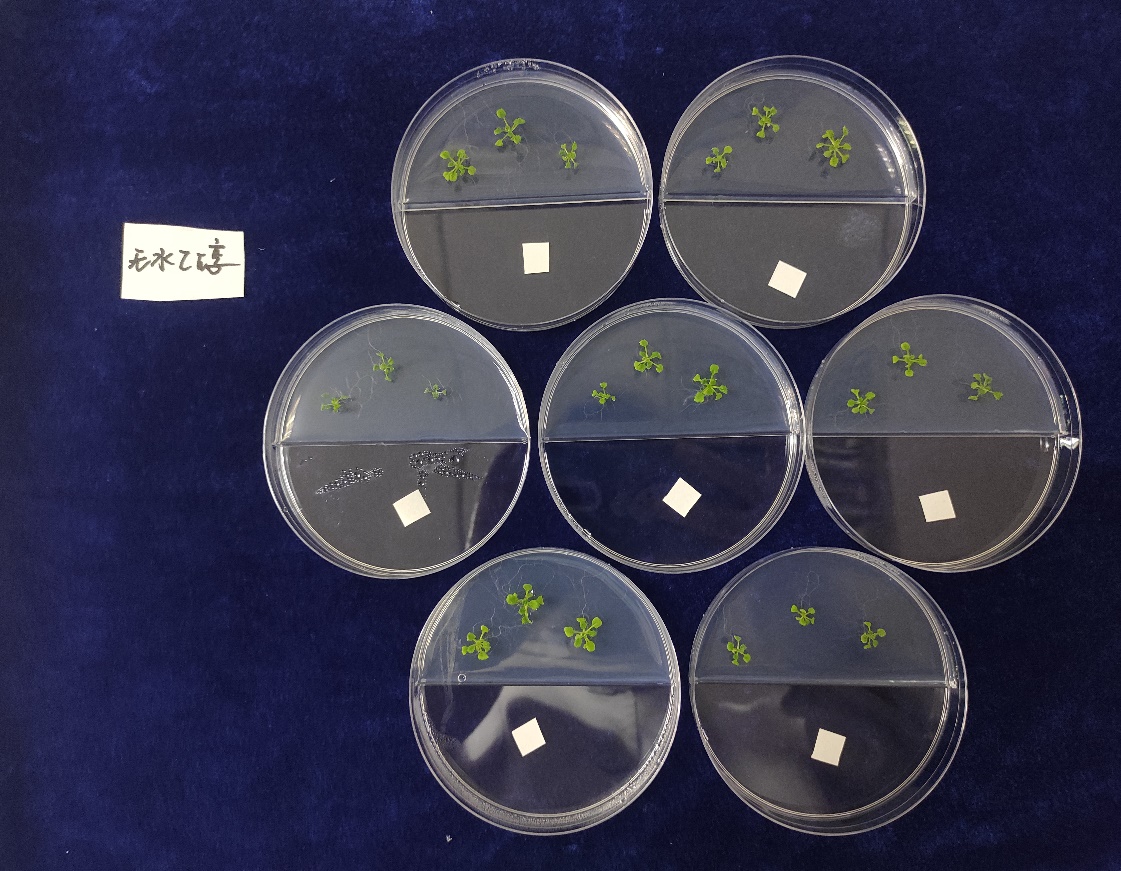


**b**


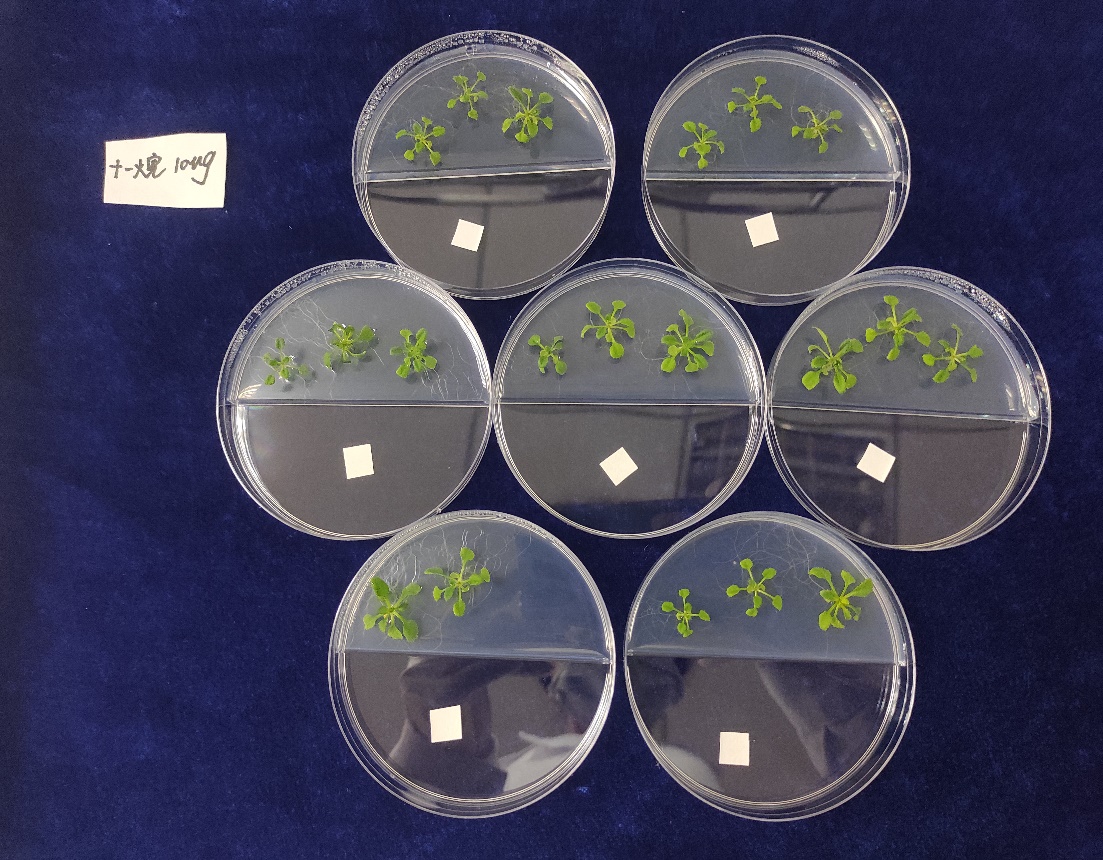


**c**


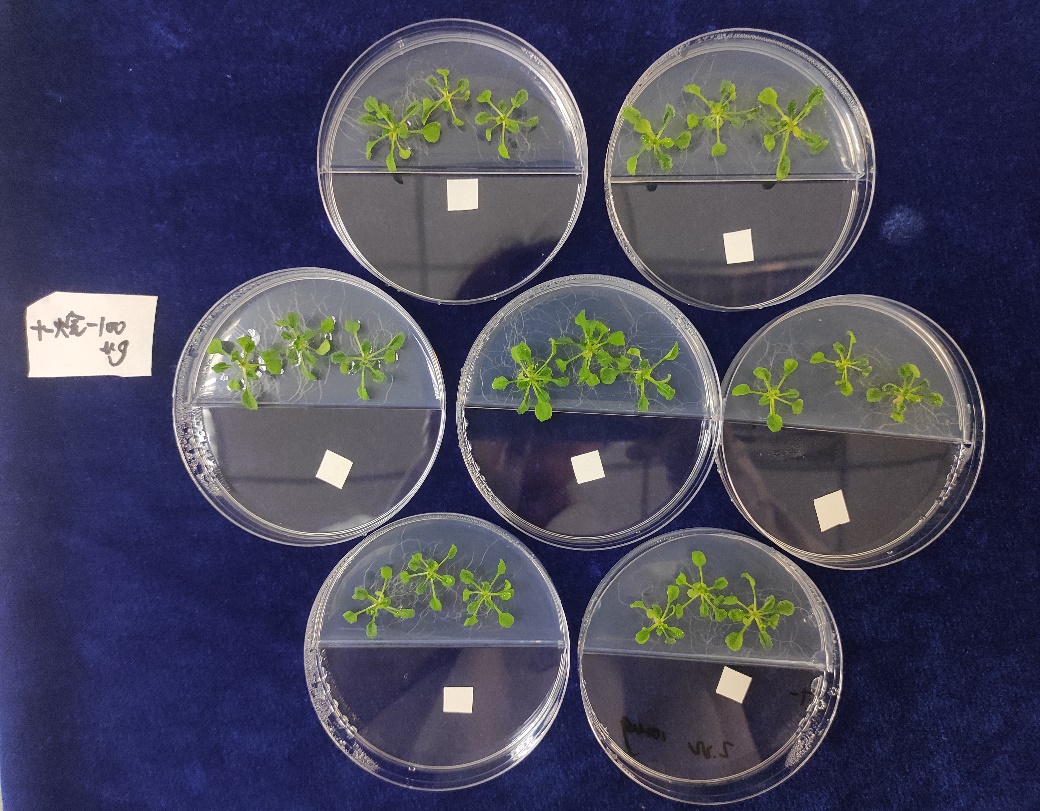


**d**


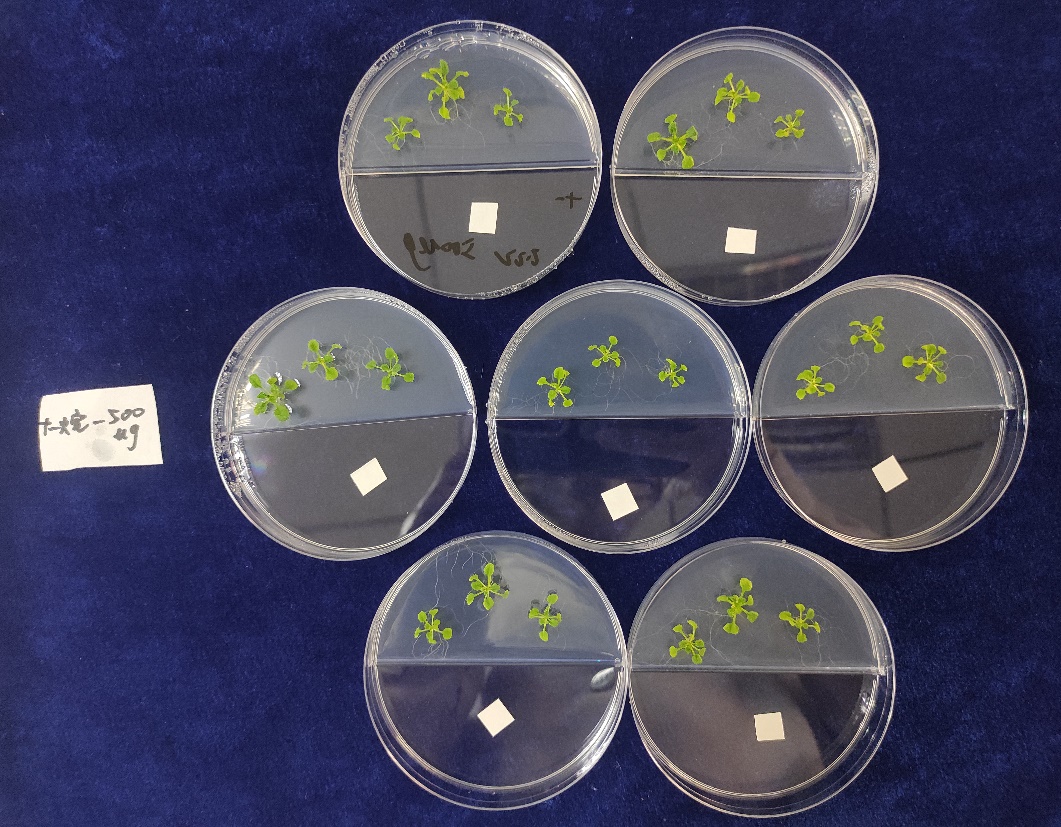


**e**


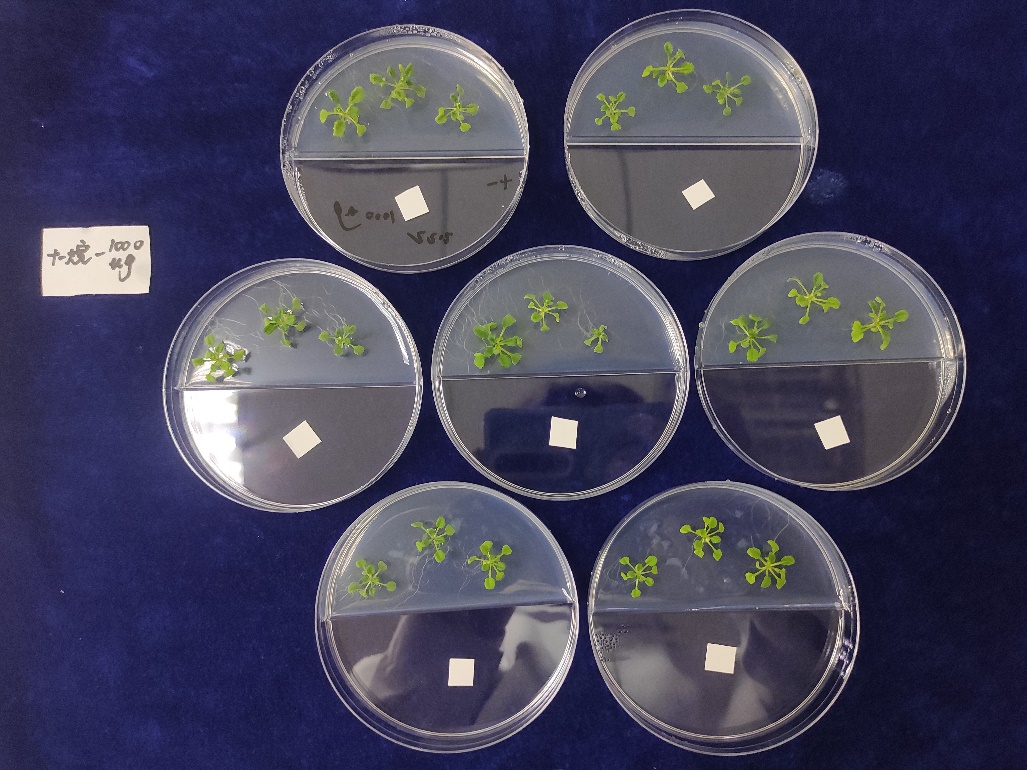


**f**


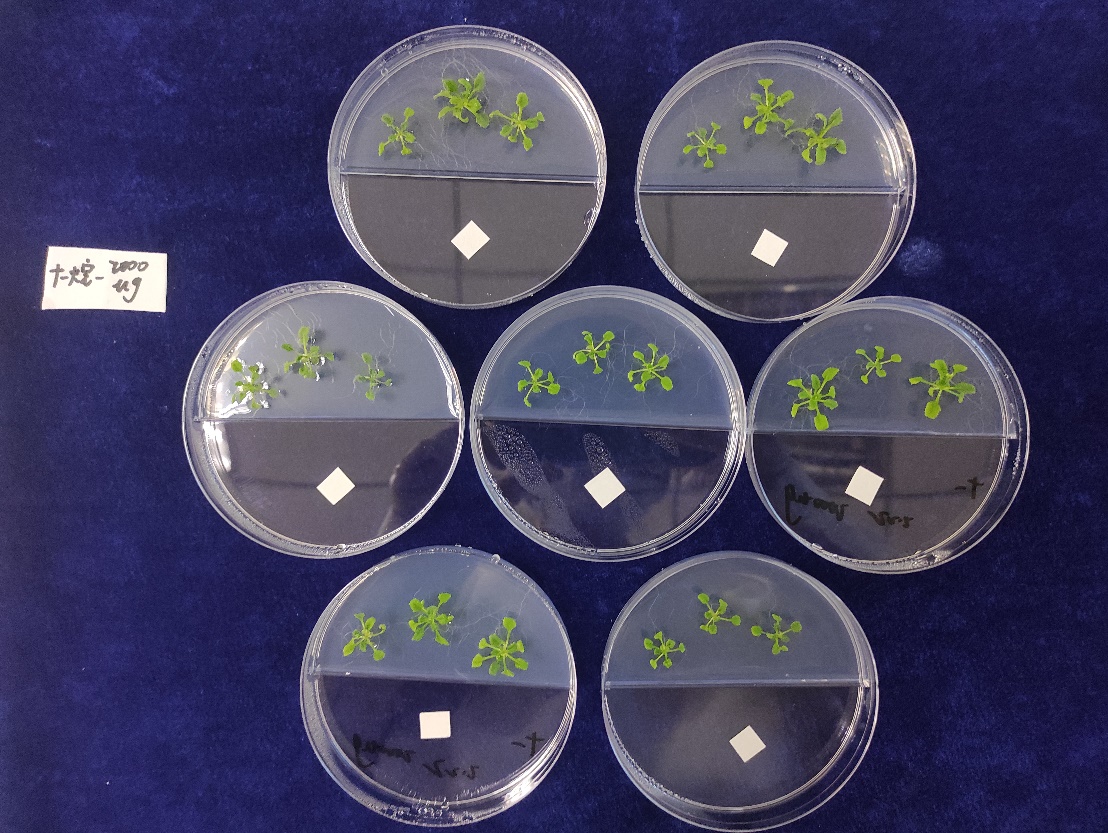


**g**


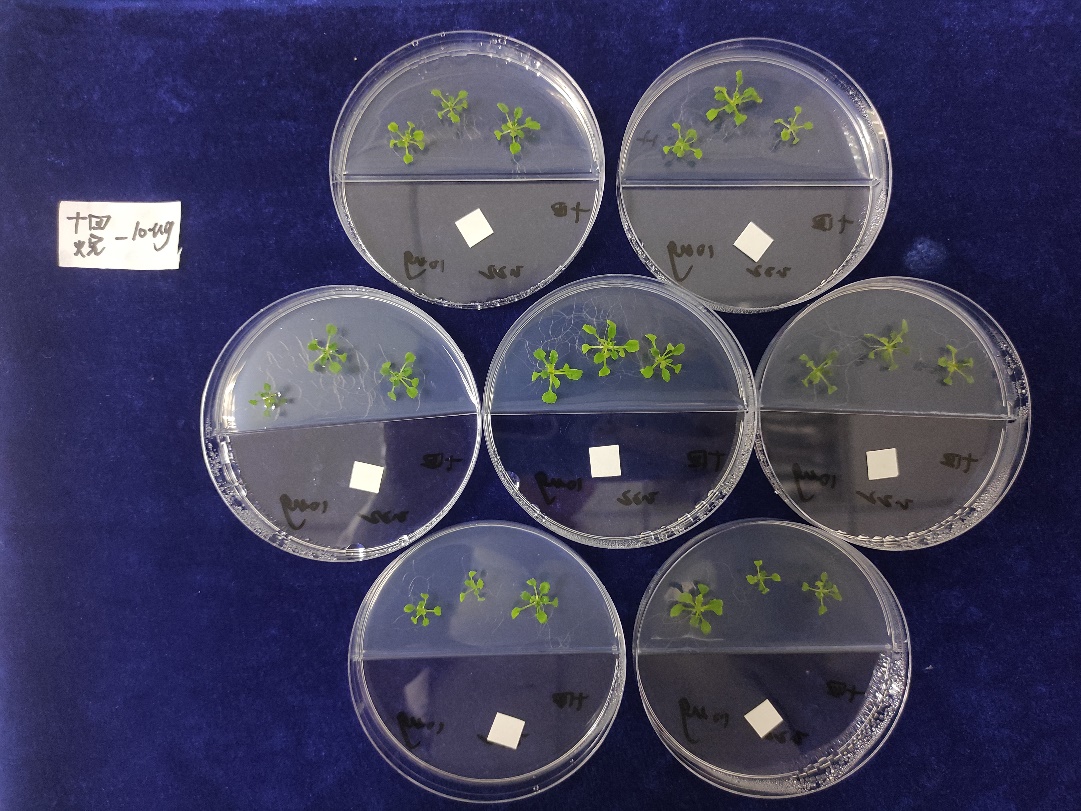


**h**


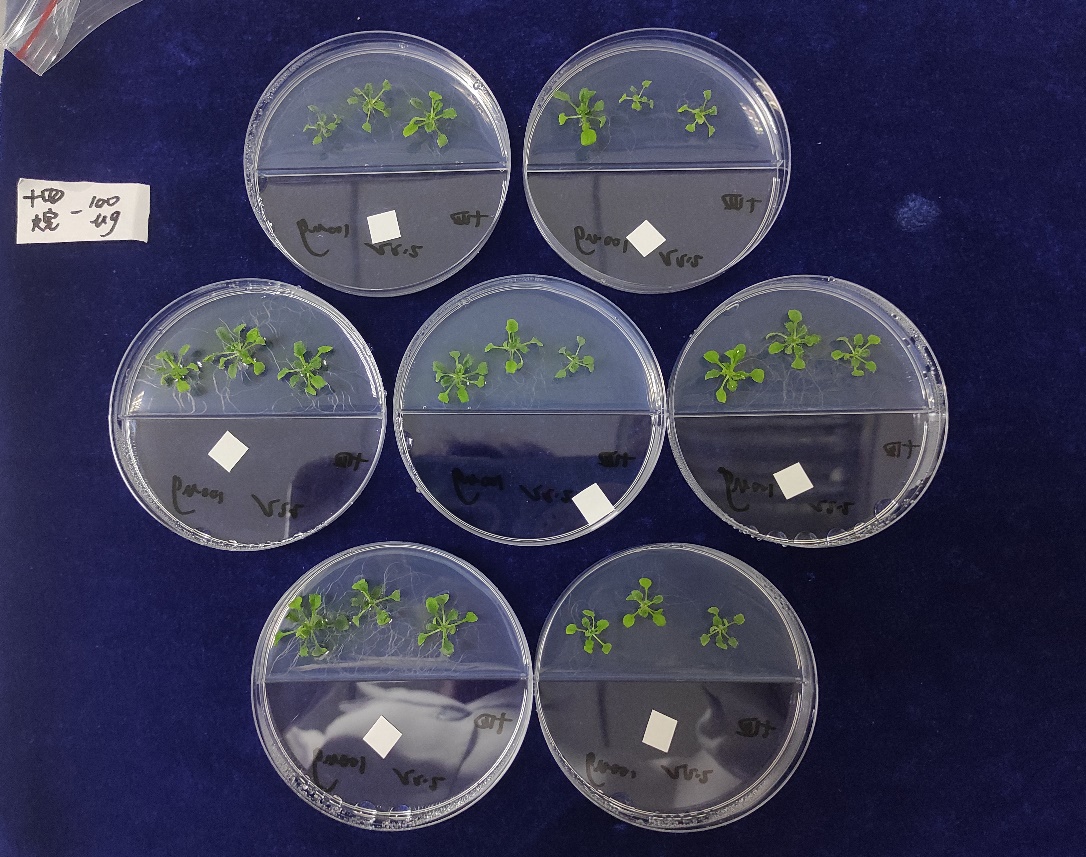


**i**


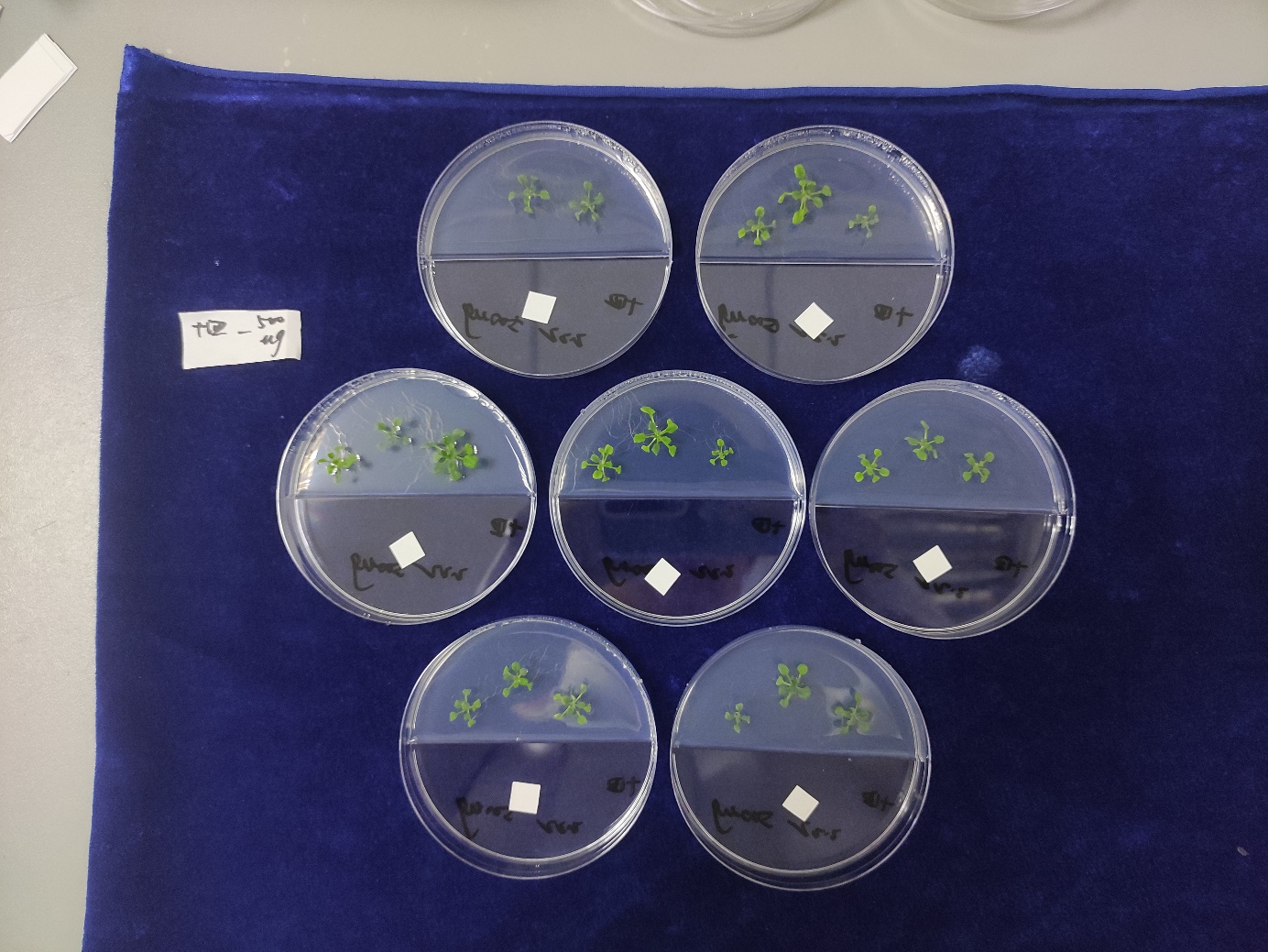


**j**


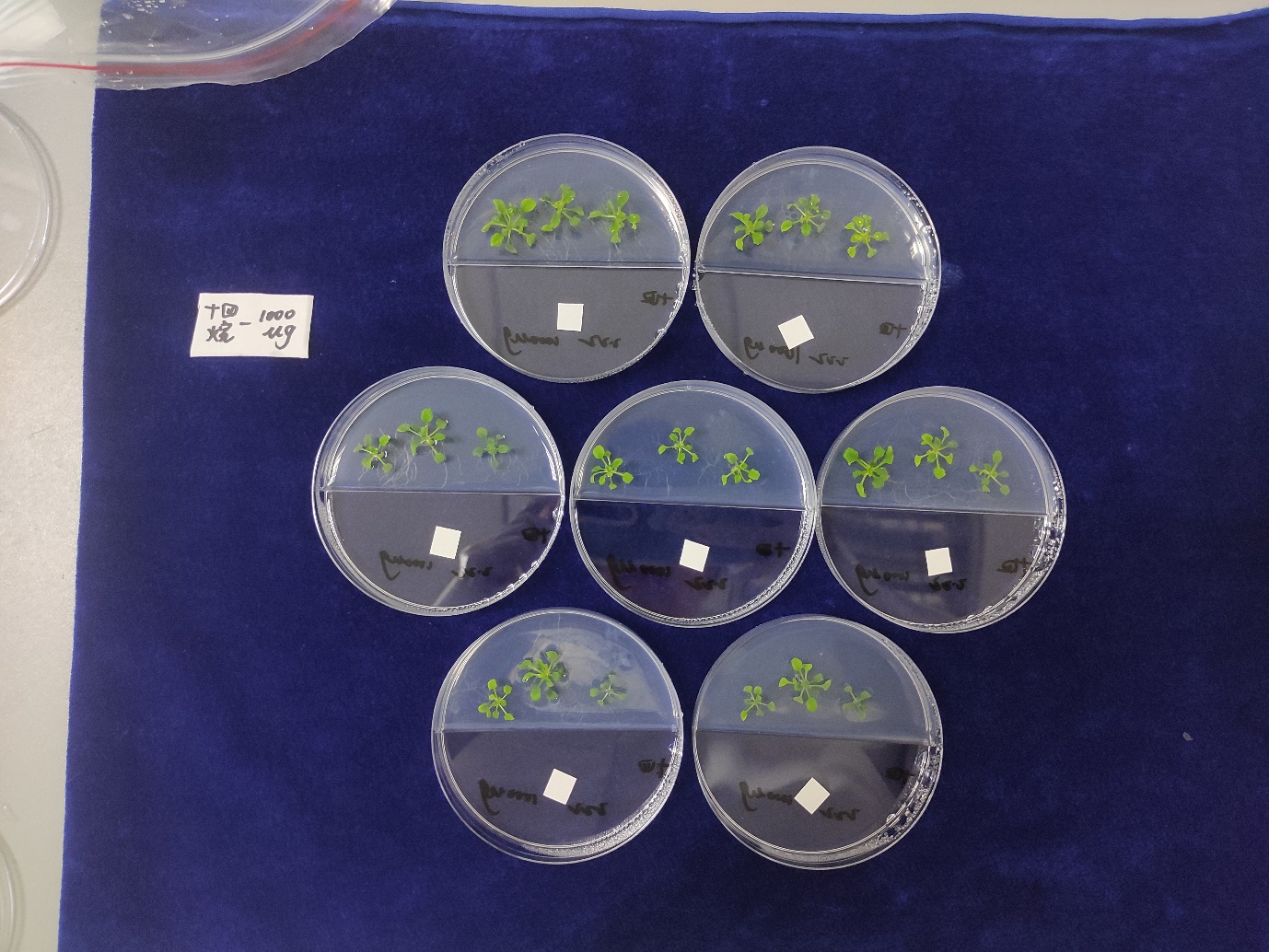


**k**


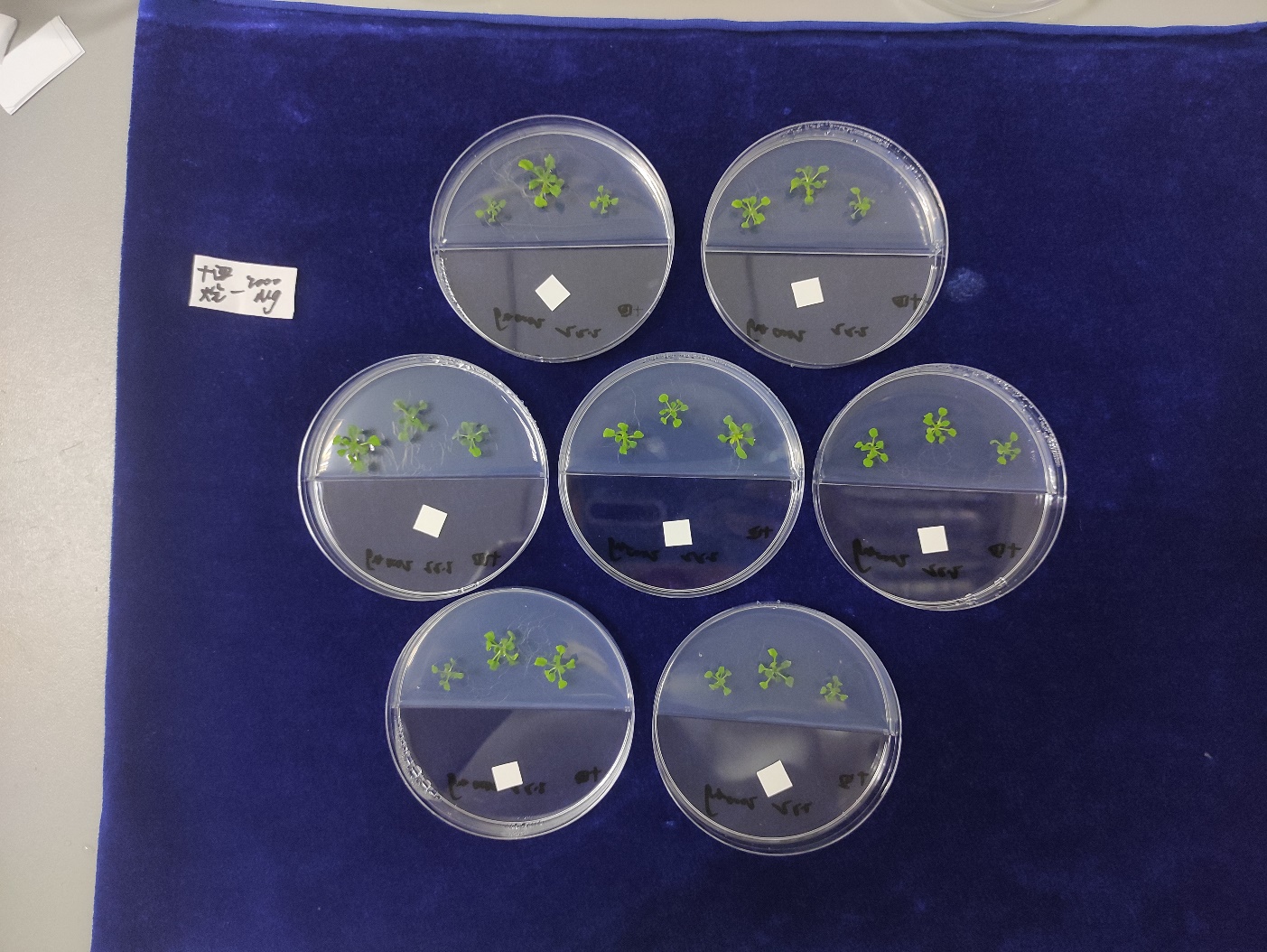


**l**


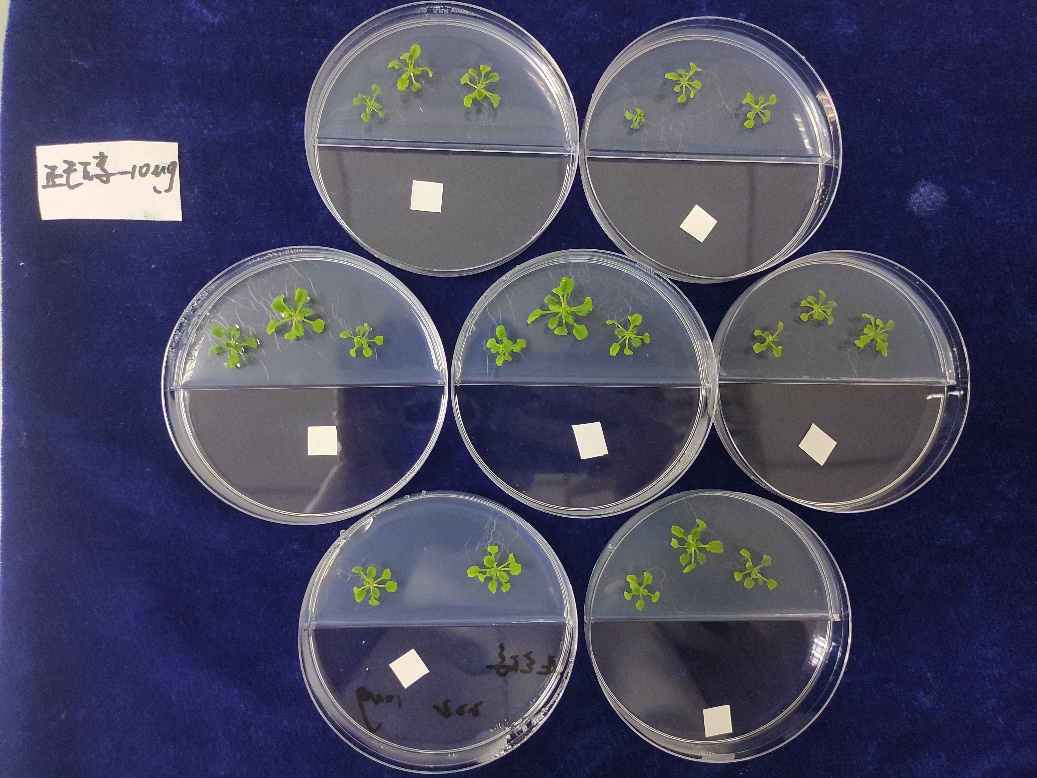


**m**


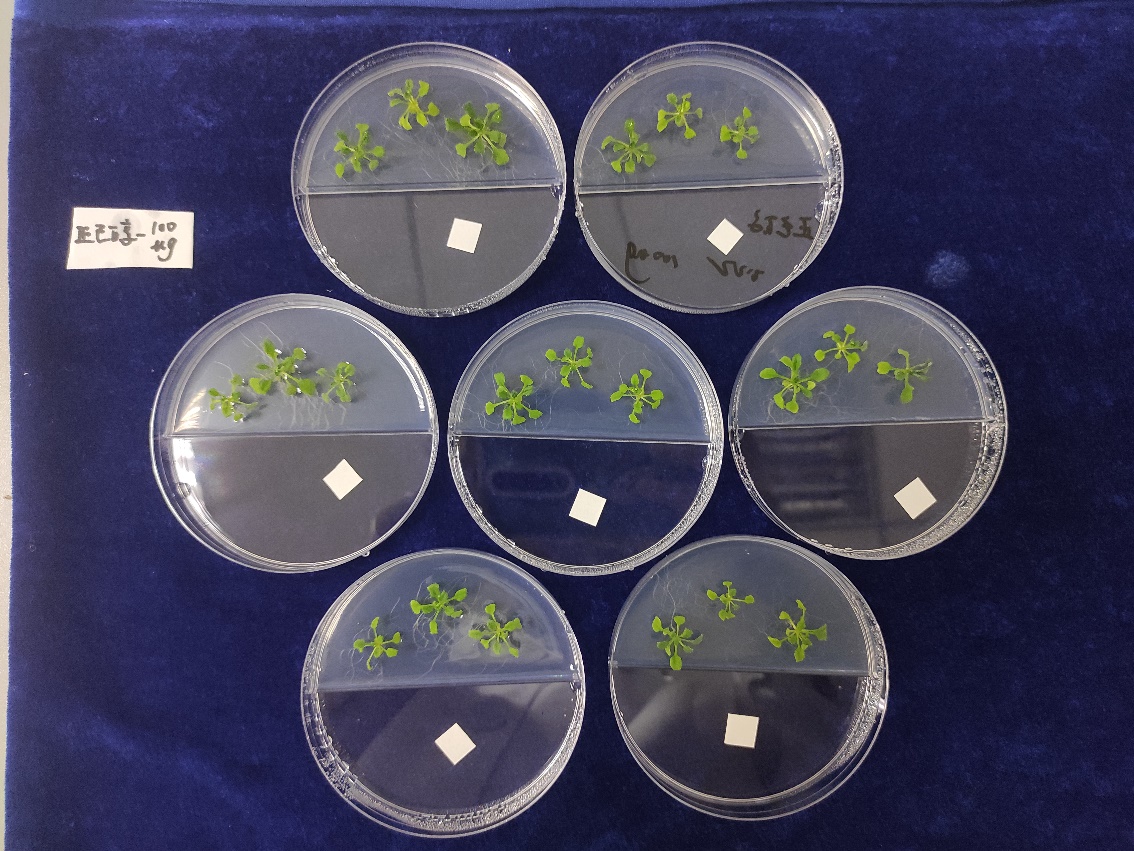


**n**


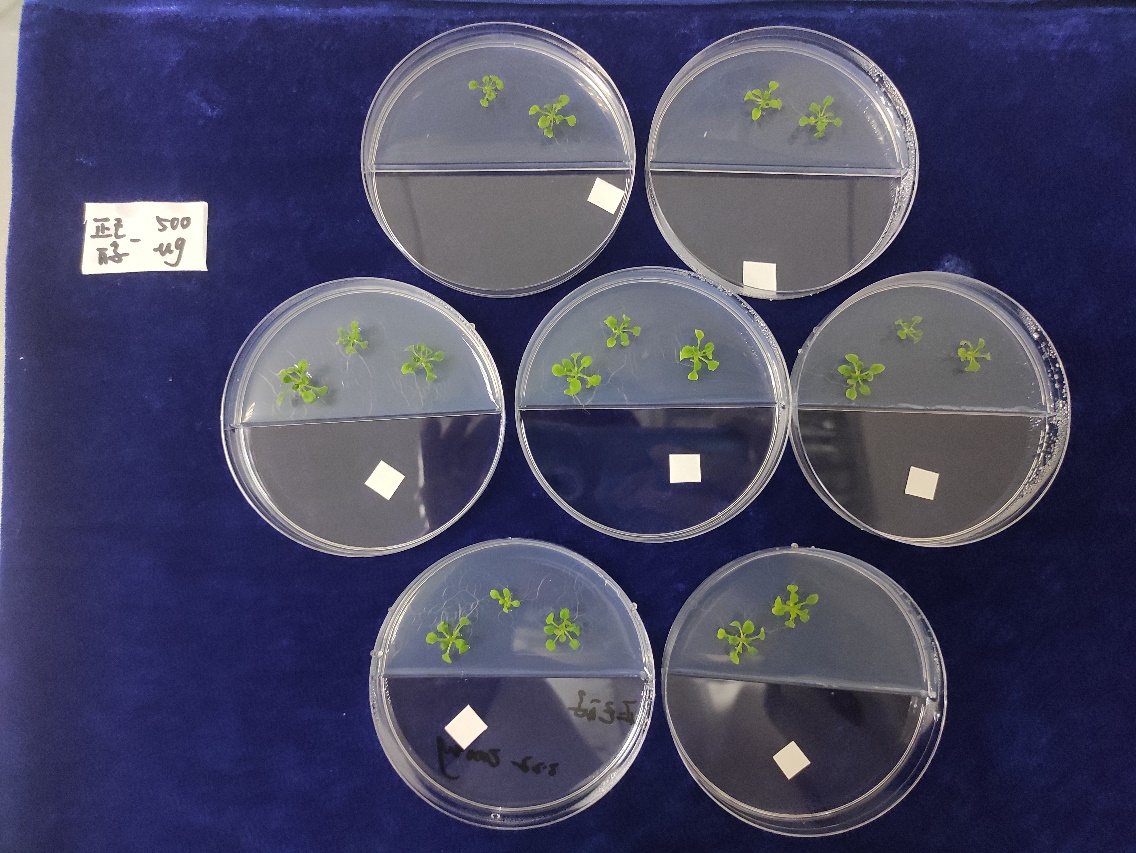


**o**


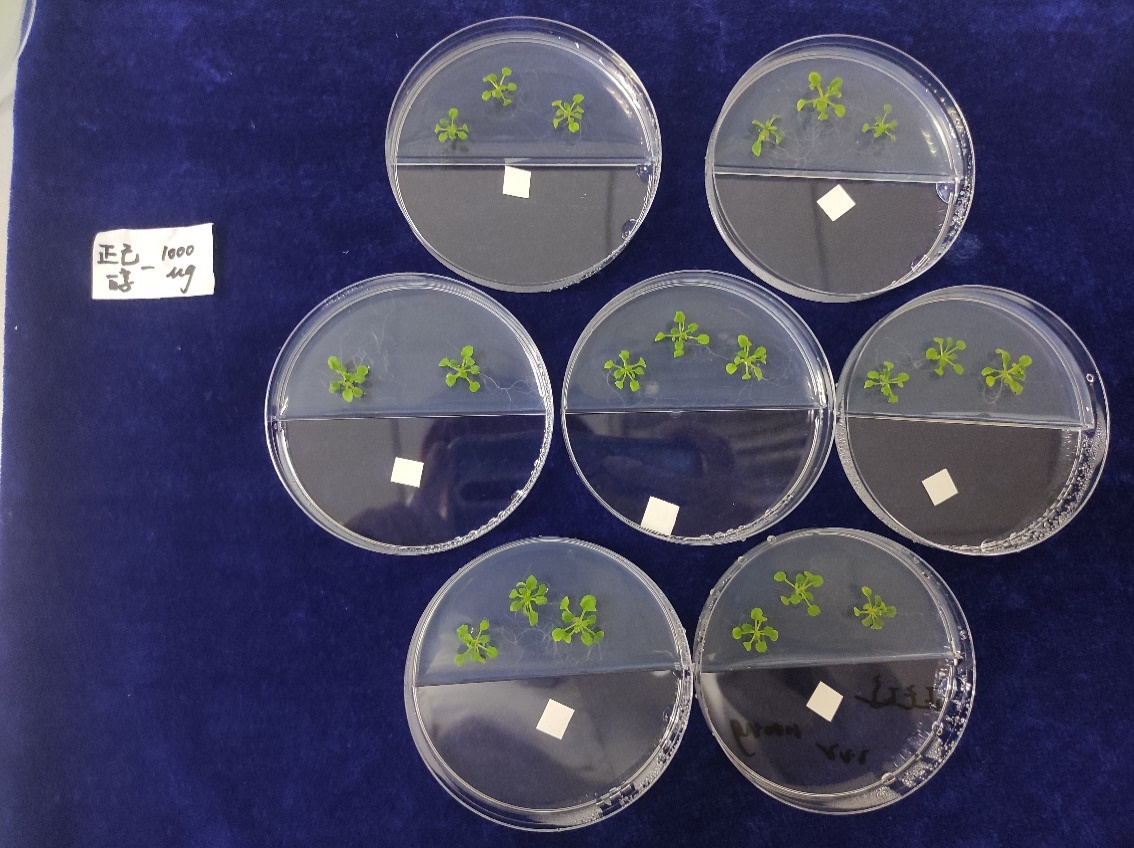


**p**


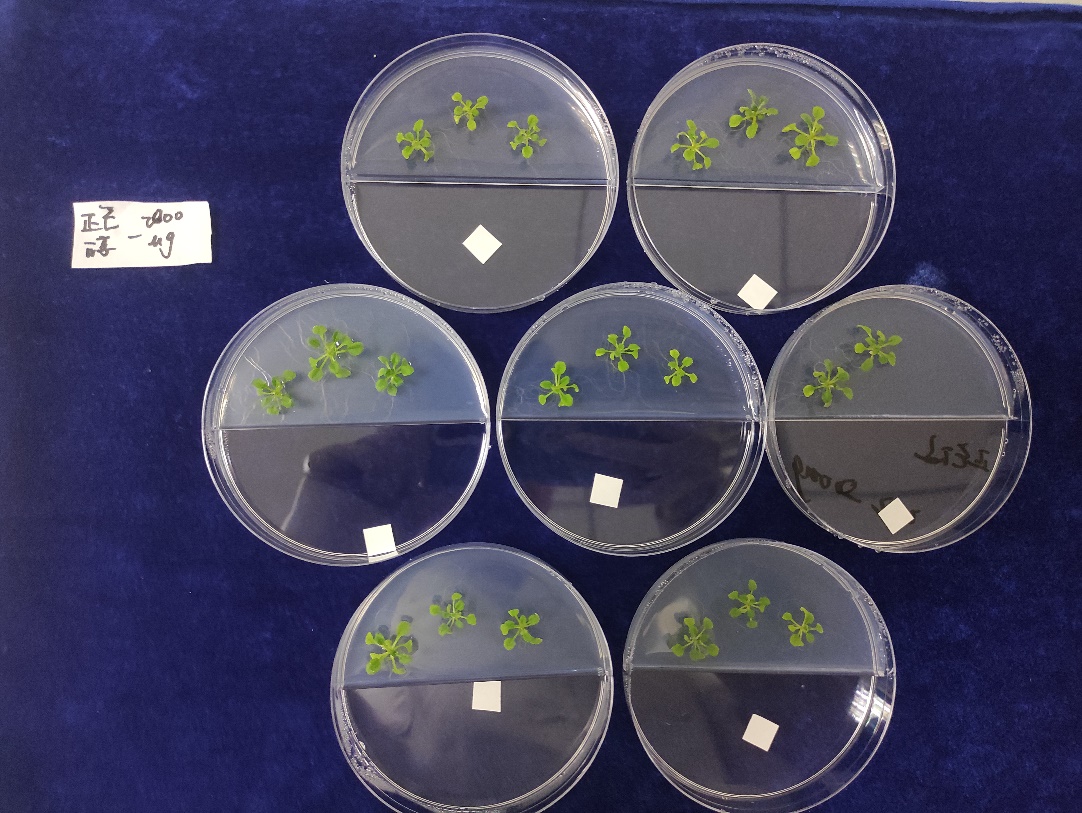


**q**

**Figure S5.** Inhibitory effect of selected VOCs on the growth of *Arabidopsis.* a: sterile water; b: ethanol; c: 10 µg hendecane; d: 100 µg hendecane; e: 500 µg hendecane; f: 1000 µg hendecane; g: 2000 µg hendecane; h: 10 µg tetradecane; i: 100 µg tetradecane; j: 500 µg tetradecane; k: 1000 µg tetradecane; l: 2000 µg tetradecane; m: 10 µg 1-hexanol; n: 100 µg 1-hexanol; o: 1-500 µg 1-hexanol; p: 1000 µg 1-hexanol; q: 2000 µg 1-hexanol.
